# Defective Gating and Proteostasis of Human ClC-1 Chloride Channel: Molecular Pathophysiology of Myotonia Congenita

**DOI:** 10.3389/fneur.2020.00076

**Published:** 2020-02-11

**Authors:** Chung-Jiuan Jeng, Ssu-Ju Fu, Chia-Ying You, Yi-Jheng Peng, Cheng-Tsung Hsiao, Tsung-Yu Chen, Chih-Yung Tang

**Affiliations:** ^1^Institute of Anatomy and Cell Biology, School of Medicine, National Yang-Ming University, Taipei, Taiwan; ^2^Brain Research Center, National Yang-Ming University, Taipei, Taiwan; ^3^Department of Physiology, College of Medicine, National Taiwan University, Taipei, Taiwan; ^4^Department of Neurology, Taipei Veterans General Hospital, Taipei, Taiwan; ^5^Center for Neuroscience, University of California, Davis, Davis, CA, United States; ^6^College of Medicine, Graduate Institute of Brain and Mind Sciences, National Taiwan University, Taipei, Taiwan

**Keywords:** skeletal muscle, genetic disease, mutation, channelopathy, protein quality control, protein degradation, membrane trafficking, proteostasis network

## Abstract

The voltage-dependent ClC-1 chloride channel, whose open probability increases with membrane potential depolarization, belongs to the superfamily of CLC channels/transporters. ClC-1 is almost exclusively expressed in skeletal muscles and is essential for stabilizing the excitability of muscle membranes. Elucidation of the molecular structures of human ClC-1 and several CLC homologs provides important insight to the gating and ion permeation mechanisms of this chloride channel. Mutations in the human *CLCN1* gene, which encodes the ClC-1 channel, are associated with a hereditary skeletal muscle disease, myotonia congenita. Most disease-causing *CLCN1* mutations lead to loss-of-function phenotypes in the ClC-1 channel and thus increase membrane excitability in skeletal muscles, consequently manifesting as delayed relaxations following voluntary muscle contractions in myotonic subjects. The inheritance pattern of myotonia congenita can be autosomal dominant (Thomsen type) or recessive (Becker type). To date over 200 myotonia-associated ClC-1 mutations have been identified, which are scattered throughout the entire protein sequence. The dominant inheritance pattern of some myotonia mutations may be explained by a dominant-negative effect on ClC-1 channel gating. For many other myotonia mutations, however, no clear relationship can be established between the inheritance pattern and the location of the mutation in the ClC-1 protein. Emerging evidence indicates that the effects of some mutations may entail impaired ClC-1 protein homeostasis (proteostasis). Proteostasis of membrane proteins comprises of biogenesis at the endoplasmic reticulum (ER), trafficking to the surface membrane, and protein turn-over at the plasma membrane. Maintenance of proteostasis requires the coordination of a wide variety of different molecular chaperones and protein quality control factors. A number of regulatory molecules have recently been shown to contribute to post-translational modifications of ClC-1 and play critical roles in the ER quality control, membrane trafficking, and peripheral quality control of this chloride channel. Further illumination of the mechanisms of ClC-1 proteostasis network will enhance our understanding of the molecular pathophysiology of myotonia congenita, and may also bring to light novel therapeutic targets for skeletal muscle dysfunction caused by myotonia and other pathological conditions.

## Introduction

Myotonia is characterized as delayed muscle relaxation following voluntary or induced (e.g., electrical or mechanical stimulations) contraction, indicating hyperexcitability in the plasma membrane of skeletal muscle fibers. In myotonia associated with muscle dystrophies (myotonic dystrophy), trinucleotide and tetranucleotide repeat mutations in the *DMPK* and *ZNF9*/*CNBP* genes, respectively, lead to progressive dysfunction in multiple systems including the heart, brain, eye, and skeletal muscle (1–3). Non-dystrophic myotonias, in contrast, result from mutations in the genes encoding muscle ion channels, leading to electrical hyperexcitation and excessive contraction of skeletal muscles (4–7).

Disease arising from ion channel disorders is commonly known as channelopathy. One of the channelopathies associated with non-dystrophic myotonia concerns a chloride (Cl^−^) channel critical for the function of skeletal muscles, the voltage-dependent ClC-1 Cl^−^ channel. Mutations in the human *CLCN1* gene lead to involuntary muscle contractions caused by anomalous sarcolemmal action potentials, clinically known as myotonia congenita (8–11). The worldwide prevalence rate of myotonia congenita is estimated to be 1:100,000, with a higher prevalence (about 1:10,000) in northern Scandinavia (12–14). To date, over 200 distinct mutations in the human ClC-1 protein have been linked to myotonia congenita (9, 15). This review aims to provide an up-to-date overview of the mechanisms of disease-related disruption of ClC-1 channel function. Specifically, we will address the significance of impaired ClC-1 protein stability and trafficking in the molecular pathophysiology of myotonia congenita.

## Structure and Function of the ClC-1 Channel

The ClC-1 protein is a member of the CLC channel/transporter superfamily. The mammalian CLC family consists of nine members, with four (ClC-1, ClC-2, ClC-Ka, ClC-Kb) Cl^−^ channels predominantly residing in the plasma membrane, and the rest (ClC-3, ClC-4, ClC-5, ClC-6, ClC-7) Cl^−^/H^+^ antiporters (counter transporters) mostly located in intracellular organelles (16–20). The structural detail of the CLC channels/transporters is made available by latest breakthroughs in obtaining the crystal or cryogenic electron microscopy (cryo-EM) structures of various CLC proteins, including bacterial ClC-ec1, thermophilic algal CmClC, bovine ClC-K, and most recently human ClC-1 (21–26). Together they provide important insight to the gating and ion permeation mechanisms of the ClC-1 channel.

**Figure 1:**
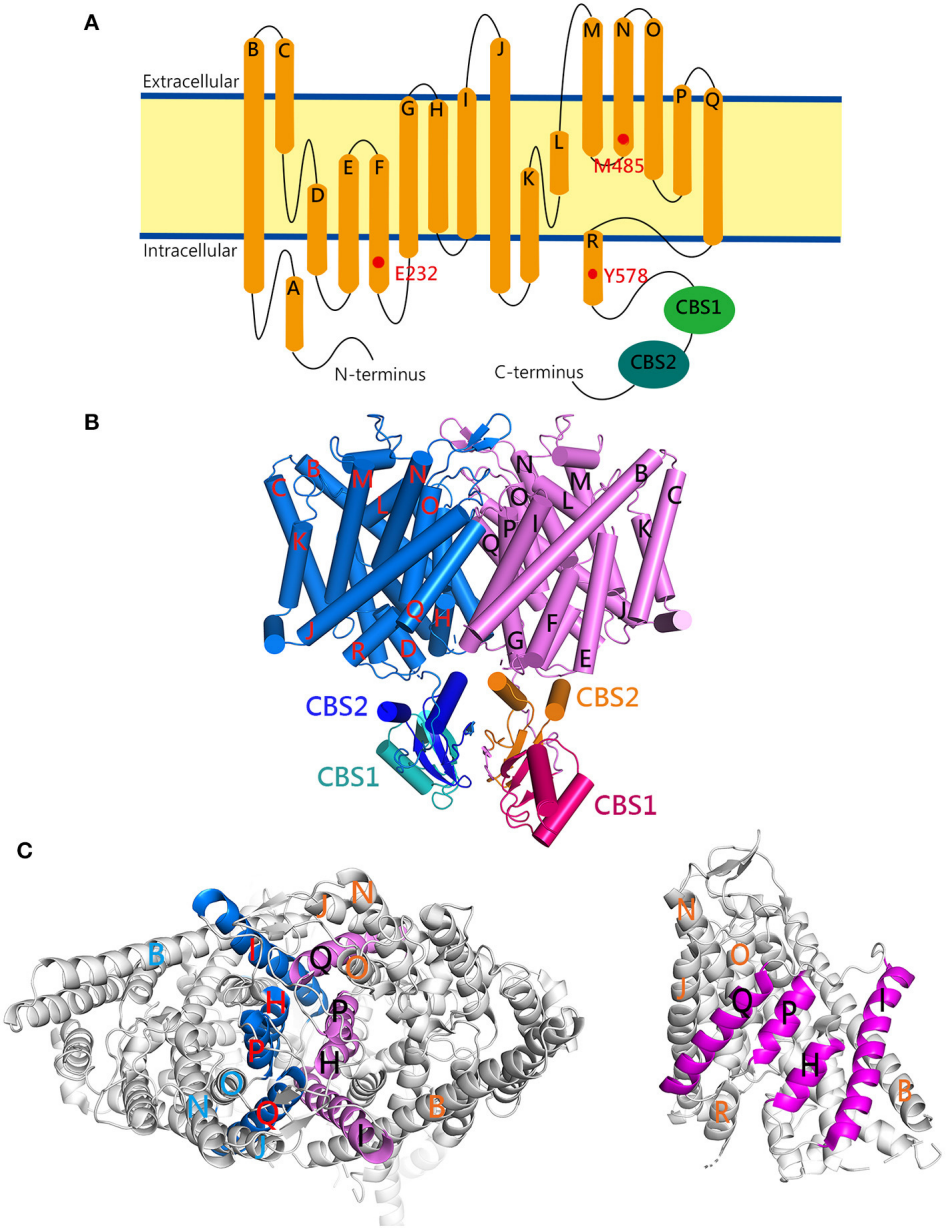
The cryo-EM structure of the human ClC-1 channel. **(A)** Membrane topology of the ClC-1 subunit. The α-helices (A–R) are represented as cylinders. The locations of three pore-lining residues (E232, M485, Y578) and two cystathionine β-synthase (CBS) domains (CBS1, CBS2) are indicated. **(B)** Lateral view of the ClC-1 dimer (PDB code: 6QVC; presented using Pymol). The α-helices are shown as cylinders. The transmembrane portions of the two subunits in the dimer are colored in blue and magenta, respectively. Also highlighted are the CBS domains in the cytoplasmic carboxyl-terminal portion of each subunit. **(C)** The dimer interface of ClC-1. The interface-forming helices (H, I, P, Q) are drawn as colored ribbons. (Left) The ClC-1 dimer is viewed from the extracellular side. (Right) The ClC-1 subunit is viewed from the dimer interface of the opposing subunit.

The human ClC-1 channel is a transmembrane protein consisting of 988 amino acids (a.a.; with an apparent molecular weight of about 120 kDa), generally divided into the amino (N)-terminal transmembrane portion (up to about 590 a.a.) and the carboxyl (C)-terminal cytoplasmic portion (Figure 1A). The transmembrane portion of the human ClC-1 protein is composed of 18 α-helices (helices A–R), with 17 (helices B–R) membrane-associated. Most of these helices are not perpendicular to the plasma membrane, but rather notably tilted. Interestingly, many of these helices fail to span the entire width of the lipid membrane. Furthermore, the cytoplasmic C-terminal portion also contains two tandem helical regions, the cystathionine β-synthase (CBS) domains (CBS1 and CBS2), which fold into an ATP-binding site (27).

Both functional and structural analyses support the notion that, like the other members of the CLC protein family, a functional ClC-1 channel comprises of a homodimeric structure [Figure 1B; (21–26, 28–32)]. The H, I, P, and Q helices in each ClC-1 subunit constitute the subunit interface between the two protomers (the dimer interface) (Figure 1C). Moreover, within each subunit of the ClC-1 homodimer, there is a separate ion-conducting pore (mainly formed by residues located at helices D, F, N, and R) known as the protopore. In other words, the ion-conducting pore of ClC-1 is entirely contained within each subunit of the dimer, and a functional ClC-1 channel thus harbors two protopores.

Consistent with the functional properties originally inferred from single-channel recordings of its fish homolog (the *Torpedo* ClC-0 channel), the opening of ClC-1 channel entails three different conductance levels that correspond to the opening of two independent ion-conducting pores, a phenomenon coined the “double-barreled” single-channel behavior (16, 28–33). This notion is further supported by cryo-EM analyses showing the presence of two protopores in a human ClC-1 homodimer (24, 25). As in all CLC channels, the opening and closing (gating) of the two protopores in ClC-1 is controlled by two distinct mechanisms (16, 20): (i) the “fast-gate” that controls the opening and closing of each protopore independently from the partner fast-gate, and (ii) the “common-gate” that controls the two protopores simultaneously. Thus, activation of the ClC-1 ion-conducting pathway requires the opening of both the common-gate and the fast-gate.

The opening kinetics of the ClC-1 fast-gate accelerates significantly in response to membrane depolarization (33–35). This gating mechanism is fast enough to counteract the depolarization conferred by voltage-gated sodium (Na^+^) channels during an action potential, and is thus important for regulating skeletal muscle contraction. Besides the control by membrane potential, the fast-gate is also subject to modulation by Cl^−^ and H^+^ (30, 33–36). Similar to voltage-gated cation channels, the open probability (*P*_o_) of ClC-1 fast-gating is higher at more depolarized membrane potentials. Unlike voltage-gated cation channels, however, the ClC-1 protein does not seem to contain any transmembrane segment serving as the “voltage sensor.” Rather, like ClC-0, the voltage-dependent activation of the fast-gate of ClC-1 may also arise from the coupling of Cl^−^ transport with the gating process (34, 37, 38). This gating-permeation coupling mechanism is supported by two findings: reducing the extracellular Cl^−^ concentration shifts the steady-state voltage dependence of *P*_o_ (*P*_o_-V curve) of ClC-1 fast-gating toward a more depolarized membrane potential, and extracellular Cl^−^ raises the *P*_o_ by increasing the opening rate of the ClC-1 fast-gate (33–35). Together, these observations can be explained by a Cl^−^-gating model in which the binding of Cl^−^ to the protopore opens the ClC-1 fast-gate, and Cl^−^ crossing the membrane electric field provides the fundamental mechanism for the observed voltage dependence (16). Importantly, the glutamate-232 residue (E232), located at the beginning of helix F of human ClC-1 (Figure 1A), may protrude its negatively-charged side-chain into the Cl^−^-permeation pathway, and serve as the gate that controls each individual protopore (16, 23–25, 39–41). Other notable pore-lining residues in the human ClC-1 include methionine 485 (M485; located at helix N) and tyrosine 578 (Y578; located at helix R) (Figure 1A). The former is located at the narrowest constriction at the extracellular opening of the pore and may serve as a hydrophobic barrier, while the latter constitutes a Cl^−^-binding site at the intracellular opening of the pore and forms part of the selectivity filter (24, 25).

The opening rate and *P*_o_ of the ClC-1 common-gate (also known as the slow-gate) are voltage-dependent as well, both becoming higher at more depolarized membrane potentials (33, 35, 42). Nevertheless, the detailed mechanism of the common-gating remains obscure (20). Formation of heterodimeric CLC channels comprising ClC-0 and ClC-1 or ClC-2 concatemers results in the loss of the ClC-0 common-gating, but without detectably affecting single channel conductance of individual ClC-0, ClC-1, and ClC-2 protopores (32). Interestingly, dissociation of the common-gating was observed in heterodimeric ClC-1-ClC-2 concatemers (43). Moreover, mutations of several residues located at or close to the dimer interface lead to significant alterations of the ClC-1 common-gating (42, 44–46). Together these results suggest that the mechanism of the common-gating entails the relative motion of the two channel subunits (i.e., inter-subunit interactions). In ClC-0, the common-gating may additionally involve the movement of the C-terminal cytoplasmic domain (47). Consistent with this idea, nucleotides (such as ATP) binding to the C-terminal cytoplasmic CBS domains seems to preclude the opening of the ClC-1 common-gate (27, 48–50). This may involve interactions between the CBS2 domain and the intracellular loop connecting helices D and E (24). Finally, the pore-lining E232 and Y578 have also been implicated in the ClC-1 common-gating (51).

Despite the presence of low-level expression in some other tissues, the ClC-1 channel is virtually exclusively expressed in skeletal muscles (52, 53). While multiple types of Cl^−^ channels exist in skeletal muscles, the ClC-1 channel is the most abundant (54–56). In most adult mammalian cells, the extracellular Cl^−^ concentration is significantly higher than its intracellular counterpart, leading to a negative Cl^−^ equilibrium potential (57). The physiological significance of the ClC-1 channel is further highlighted by the finding that Cl^−^ channel conductance may contribute up to 80% of the resting membrane conductance of skeletal muscle (58–60), and that Cl^−^ conductance is essential for preventing excessive firing of muscle action potentials (61). In addition to the sarcolemma, a significant Cl^−^ conductance is also present in the transverse-tubule system of skeletal muscle (59, 62–64). Although the precise subcellular localization pattern of ClC-1 in skeletal muscles remains contentious (56, 65–70), it is likely that ClC-1 is important for maintaining an effective Cl^−^ homeostasis system in both the sarcolemma and the transverse-tubule system. Taken together, activation of the ClC-1 channel is crucial for ensuring electrical stability of skeletal muscles by resetting membrane excitability after firing an action potential.

Several lines of evidence suggest that regulation of skeletal muscle fatigue involves alteration of ClC-1 channel activation (62, 71–74). During exercise, intensive firing of action potentials associated with active muscle contractions may result in extracellular accumulation of potassium (K^+^) ions, which in turn would depolarize muscle membrane potential and thereby induce slow inactivation of voltage-gated Na^+^ channels. Given that a sufficient inward Na^+^ current is required for adequate firing of action potentials, the reduction of the amount of active voltage-gated Na^+^ channels could disrupt the efficiency of excitation-contraction coupling in skeletal muscles and consequently lead to muscle fatigue. Furthermore, intensive exercise may cause muscle acidosis (74–76) as well as elevate intracellular calcium (Ca^2+^) concentration that activates protein kinase C (PKC). Interestingly, both intracellular acidosis and PKC activation are known to inhibit ClC-1 channel activation (49, 77–79). This down-regulation of skeletal muscle membrane Cl^−^ conductance, as well as the ensuing reduction in the membrane input conductance, effectively counteracts the effect of K^+^-induced slow inactivation of Na^+^ channels, restoring muscle excitability and preventing muscle fatigue. On the other hand, in fast-twitch muscle fibers during prolonged muscle activities, the intracellular ATP level appears to be notably lowered (74, 80), which in turn reduces ATP inhibition of ClC-1 common-gating. This enhanced opening of the ClC-1 channel is expected to decrease muscle excitability and may serve to safeguard the cellular integrity of fast-twitch muscle fibers during metabolic stress (73).

## Myotonia-Associated Aberrant Gating of Human ClC-1 Channel

Consistent with its physiological role as the cardinal Cl^−^ channel in skeletal muscles, hereditary defects in the gene encoding the ClC-1 channel result in prominently reduced membrane Cl^−^ conductance, and thus significant muscle hyperexcitability (i.e., myotonia) in animals such as goats, mice, and dogs (52, 58, 61, 81–86). Over 200 mutations in the human skeletal muscle ClC-1 gene (*CLCN1*) on chromosome 7 have been linked to myotonia congenita, which can be inherited in an autosomal recessive (Becker type) or autosomal dominant (Thomsen type) manner (8–11, 15, 87, 88). In general, the recessive Becker myotonia is clinically more severe than the dominant Thomsen form. Disease-causing *CLCN1* mutations comprise of missense, non-sense, splice-site, and frameshift mutations. The majority of *CLCN1* mutations are associated with recessive inheritance, with about 20 or less causing dominant myotonia congenita. Furthermore, about 10 mutations seem to display either a recessive or a dominant pattern (dual inheritance pattern). Myotonia-causing mutations are scattered over the entire human ClC-1 protein, including the cytosolic N- and C-terminal regions and the transmembrane domains. Overall, it is impossible to predict the inheritance pattern of *CLCN1* mutations based on mutation type or mutation location.

Myotonia congenita is one of the first proven human channelopathies. A significant number of disease-causing *CLCN1* mutations manifest as loss-of-function phenotypes in the gating/permeation of the ClC-1 channel, including the absence of discernible Cl^−^ currents (non-functional), significant shifts in the *P*_o_-V curve of fast- and/or common-gating to depolarized potentials (positive shift), and an inverted voltage-dependence in activation (hyperpolarization-activated) (10, 45, 88–93). Haploinsufficiency imparted by each loss-of-function mutant allele may therefore explain the recessive inheritance pattern of myotonia congenita. Nonetheless, since many non-functional ClC-1 mutants on only one allele fail to induce myotonia in animal models (81, 82), whether haploinsufficiency contributes to dominant inheritance remains an open question. Instead it has been suggested that dominant myotonia may be due to dominant-negative effects of the mutant subunit on the wild-type (WT) counterpart in heterozygous patients (38, 94, 95). In line with this idea, many ClC-1 mutant proteins associated with recessive myotonia (e.g., truncation mutants) do not seem to exert significant dominant-negative effects, which may be attributed to their inability to associate with the WT subunit (10).

A working hypothesis on the mechanism of the dominant-negative effect of disease-causing *CLCN1* mutations is that the inheritance pattern of a mutation is decided by its functional effect on ClC-1 channel gating; mutations that impinge on the common-gating result in dominant myotonia, whereas those only changing the gating of individual protopores lead to a recessive inheritance pattern (30, 38, 95). With the exception of truncation mutations very close to the C-terminus of the human ClC-1 channel, almost all dominant mutations are missense mutations, most of which instigate significant positive-shift of the *P*_o_-V curve such that activation of the mutant channels becomes insufficient to sustain effective membrane repolarization in skeletal muscles. In other words, in the heterodimeric ClC-1 channel formed by a WT subunit and a mutant subunit associated with dominant myotonia, the common-gate controlling both protopores may be profoundly influenced by the disease-causing mutation in the mutant subunit, thereby producing a dominant-negative effect. Consistent with this notion, many mutations causing dominant myotonia notably affect the common-gating of human ClC-1 (10, 24, 42, 44, 45, 87, 88, 96). In contrast, a recessive myotonia mutation involves a missense mutation at the pore-lining M485 (M485V) that drastically changes the voltage-dependent gating and the single-channel conductance of homodimeric mutant CLC-1 channels; upon co-expression with the WT subunit, however, the M485V mutant fails to detectably affect the gating or conductance properties of heterodimeric ClC-1 channels (94).

It is important to address the fact that many disease-associated *CLCN1* mutations do yield functional Cl^−^ channels with normal gating function. For example, the biophysical properties of several recessive ClC-1 mutant channels are either only slightly different or virtually indistinguishable from those of WT channels (10, 97, 98). Likewise, some dominant ClC-1 mutants do not seem to show detectable gating defects (99–101), indicating that the foregoing hypothesis on dominant-negative mechanism is not applicable to these mutants. The association of certain *CLCN1* mutations with a dual inheritance pattern further highlights the inadequacy of the gating hypothesis (10, 95, 102, 103). Together these examples clearly demonstrate that mechanisms beyond aberrant channel gating also contribute to the molecular pathophysiology of myotonia congenita.

## Myotonia-Associated Disruption of Human ClC-1 Proteostasis

Since the skeletal muscle Cl^−^ conductance is predominantly determined by the total number of functional membrane ClC-1 channels, myotonia congenita-associated loss-of-function mutations might involve anomalous gating/permeation in individual ClC-1 channels or reduced ClC-1 protein abundance at the plasma membrane. Direct evidence supporting the latter hypothesis was first demonstrated for three disease-causing mutations located at the distal C-terminal region (A885P, R894X, and P932L): Upon heterologous expression in *Xenopus* oocytes, they all manifested significantly decreased ClC-1 protein expression at the surface membrane (104). Immunohistochemical examinations of muscle tissues from human patients carrying the R849X mutation further confirmed a dramatic loss of human ClC-1 staining in the sarcolemma (105). Importantly, despite the presence of a notable reduction in whole-cell Cl^−^ current amplitude, only A885P, but not R894X and P932L, is associated with a positive shift of the steady-state voltage-dependent activation property [Table 1; (35, 84, 104)]. Therefore, the myotonia-causing loss of muscle ClC-1 conductance in the patients can be mainly attributed to reduced surface expression of the mutant channel proteins.

**Table 1:** Gating and proteostasis properties of myotonia-causing mutant ClC-1 channels associated with reduced surface protein expression.

**Amino acid change**	**Inheritance**	***Po*-V curve**	**Proteostasis defect**	**References**
Q43R	R	Like WT	Impaired membrane trafficking	(98)
Y137D	R	Like WT	Reduced total protein level, impaired membrane trafficking	(98)
Q160H	R	Like WT	Reduced total protein level, impaired membrane trafficking	(98)
Q412P	R	Like WT	n.d.	(97)
F413C	R	Positive shift	Impaired membrane trafficking	(100, 105, 106)
A493E	D/R	Non-functional	Reduced total protein level	(107)
A531V	R	Like WT	Enhanced ERAD, impaired membrane trafficking, defective stability at the plasma membrane	(106, 108–111)
A885P	D*	Positive shift	n.d.	(84, 104)
R894X	D/R	Negative shift	Reduced total protein level	(35, 104–106)
P932L	D/R	Like WT	n.d.	(99, 104)

D, dominant; D*, dominant myotonic goat; ERAD, endoplasmic reticulum-associated degradation; n.d., mechanism not determined; P_o_-V, the steady-state voltage dependence of channel open probability; R, recessive; WT, wild-type.

**Figure 2:**
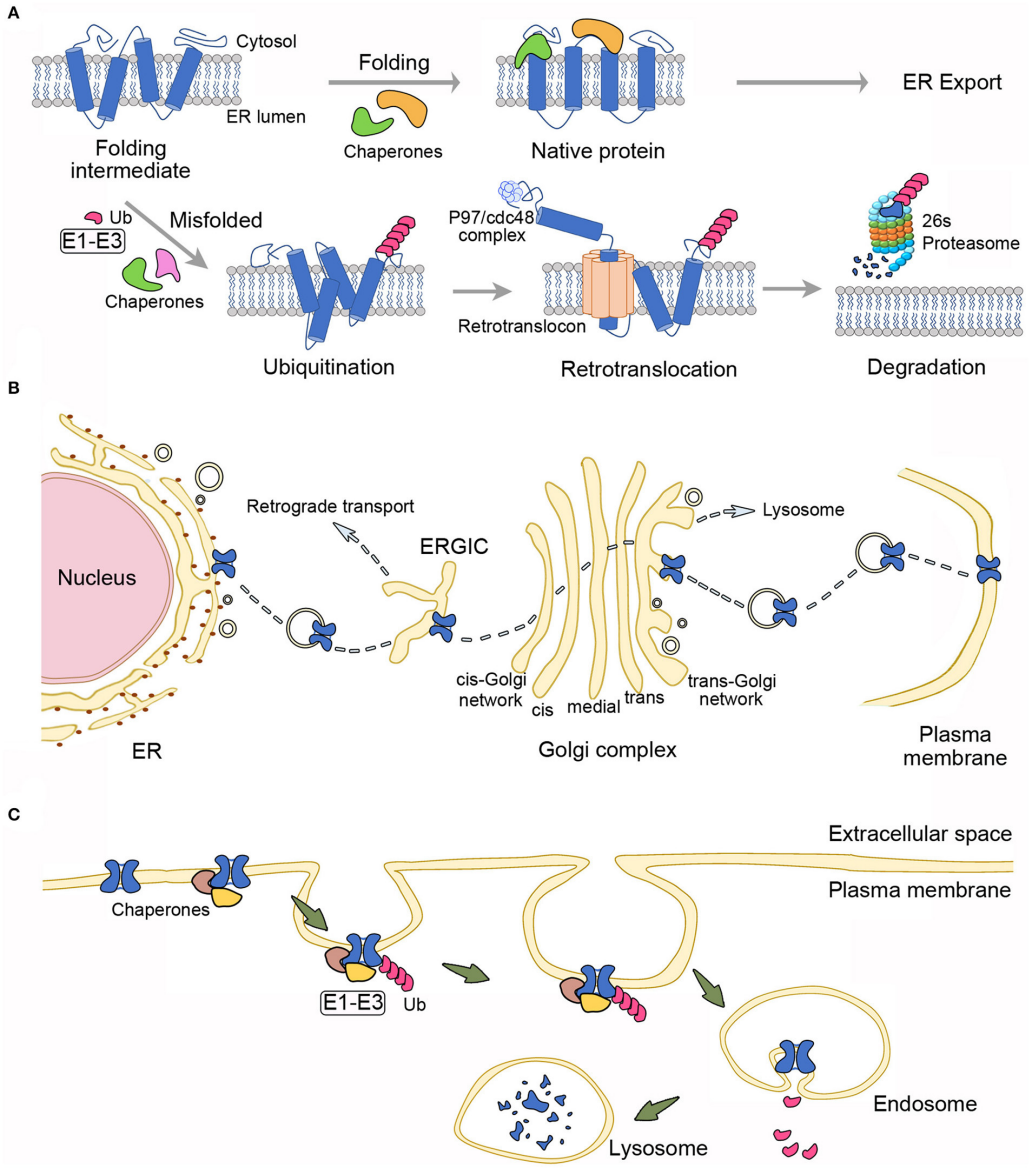
Proteostasis mechanisms governing the surface expression of membrane proteins. **(A)** Endoplasmic reticulum (ER) quality control. Protein folding at the ER is assisted by multiple molecular chaperones and cochaperones. Proteins with native folding conformation may pass the ER quality control system and are allowed to exit the ER. Chaperones/cochaperones also recognize misfolded proteins, which are subject to covalent linkage with ubiquitin (Ub) via the concerted action of three types of ubiquitination enzymes (E1–E3). The ER-associated degradation system will further target ubiquitinated proteins for retrotranslocation into the cytoplasm through the channel-like, ER membrane-localized retrotranslocon, as well as with the facilitation by the ATPase p97/Cdc48 complex. Retrotranslocated proteins are then destined for degradation by the 26S proteasome. **(B)** Membrane trafficking. Immature, native membrane proteins from the ER are packaged into transport vesicles and transferred through the ER-Golgi intermediate compartment (ERGIC) and the Golgi complex, wherein they go through further post-translational modifications. Mature proteins are eventually ushered to the plasma membrane. Misfolded proteins that escape the ER quality control system and reach the Golgi complex may still be recognized by the Golgi quality control system, followed by retrograde transport back to the ER, or antegrade transport to the lysosome. **(C)** Peripheral quality control. Molecular chaperones/cochaperones at the plasma membrane may recognize membrane proteins with conformational defects and recruit enzymes (E1–E3) for ubiquitination of the misfolded proteins, which in turn are targeted for endocytosis and lysosomal degradation.

Protein abundance is determined by the cellular maintenance of protein homeostasis (proteostasis), which controls the concentration, conformation, interaction, and subcellular localization of individual proteins (112, 113). The biological mechanisms governing proteostasis entail translational and post-translational regulations. For membrane proteins, post-translational regulation of cell surface protein density comprises of (i) protein quality control at the endoplasmic reticulum (ER quality control) (Figure 2A), (ii) trafficking to the surface membrane (membrane trafficking) (Figure 2B), and (iii) protein turn-over at the plasma membrane (peripheral quality control) [Figure 2C; (114, 115)].

Like other membrane proteins, the biogenesis of ion channels begins at the ER. After the initial translocation of a newly synthesized polypeptide into the ER membrane, channel protein folding is assisted co-translationally and post-translationally by multiple molecular chaperones and cochaperones through a series of substrate bindings and releases (116, 117). Membrane protein folding and assembly are closely monitored by the ER quality control system, composed of chaperones and associated factors, to ensure that only properly folded proteins are allowed to exit the ER [Figure 2A; (118, 119)]. Moreover, the ER quality control system recognizes and targets incorrectly folded or assembled proteins for ER-associated degradation (ERAD), which involves retrotranslocation of ubiquitinated, misfolded membrane proteins into the cytoplasm, followed with degradation by ubiquitin-proteasome machinery (120, 121). After exiting the ER, properly folded membrane proteins are packaged into ER-derived transport vesicles and then delivered to the Golgi apparatus, wherein proteins are subject to further maturation and glycosylation. Significantly, membrane proteins are also subject to a rigorous quality control at the Golgi (114, 115, 122). In general, during this membrane trafficking process, transport vesicles are progressively transferred through the ER-Golgi intermediate compartment, the *cis*-Golgi network, the Golgi stack (*cis*-, *medial*-, and *trans*-Golgi compartments), and finally to the *trans*-Golgi network, from which mature proteins are shipped to the plasma membrane [Figure 2B; (123–126)]. Emerging evidence further indicates that at the plasma membrane, misfolded membrane proteins escaped from the ER/Golgi quality control or generated in post-ER compartments are recognized by the molecular chaperones/cochaperones of the peripheral quality control system. (114, 127–129). The peripheral quality control system then removes the improperly folded proteins by ubiquitin modification, endocytosis, and subsequent trafficking to the lysosome for protein degradation (Figure 2C).

A significant number of different human disorders have been associated with proteostasis impairment that entails chronic expression of misfolded, mutant proteins with defective stability (130–132). For mutant membrane proteins with proteostasis deficiencies, the underlying molecular pathophysiological mechanisms may involve enhanced ERAD, impaired membrane trafficking, and/or defective stability at the plasma membrane (114, 125, 133, 134). Some of the well characterized proteostasis deficiencies concern the mutant Cl^−^ channels and K^+^ channels causing cystic fibrosis and long-QT syndrome, respectively (135, 136). In the case of the aforementioned myotonia congenita-associated human ClC-1 mutants A885P, R894X, and P932L, their defective surface protein density appears to arise from reduced total protein levels and/or impaired membrane trafficking (104). The precise mechanism underlying their proteostasis impairment, however, remains elusive.

**Figure 3:**
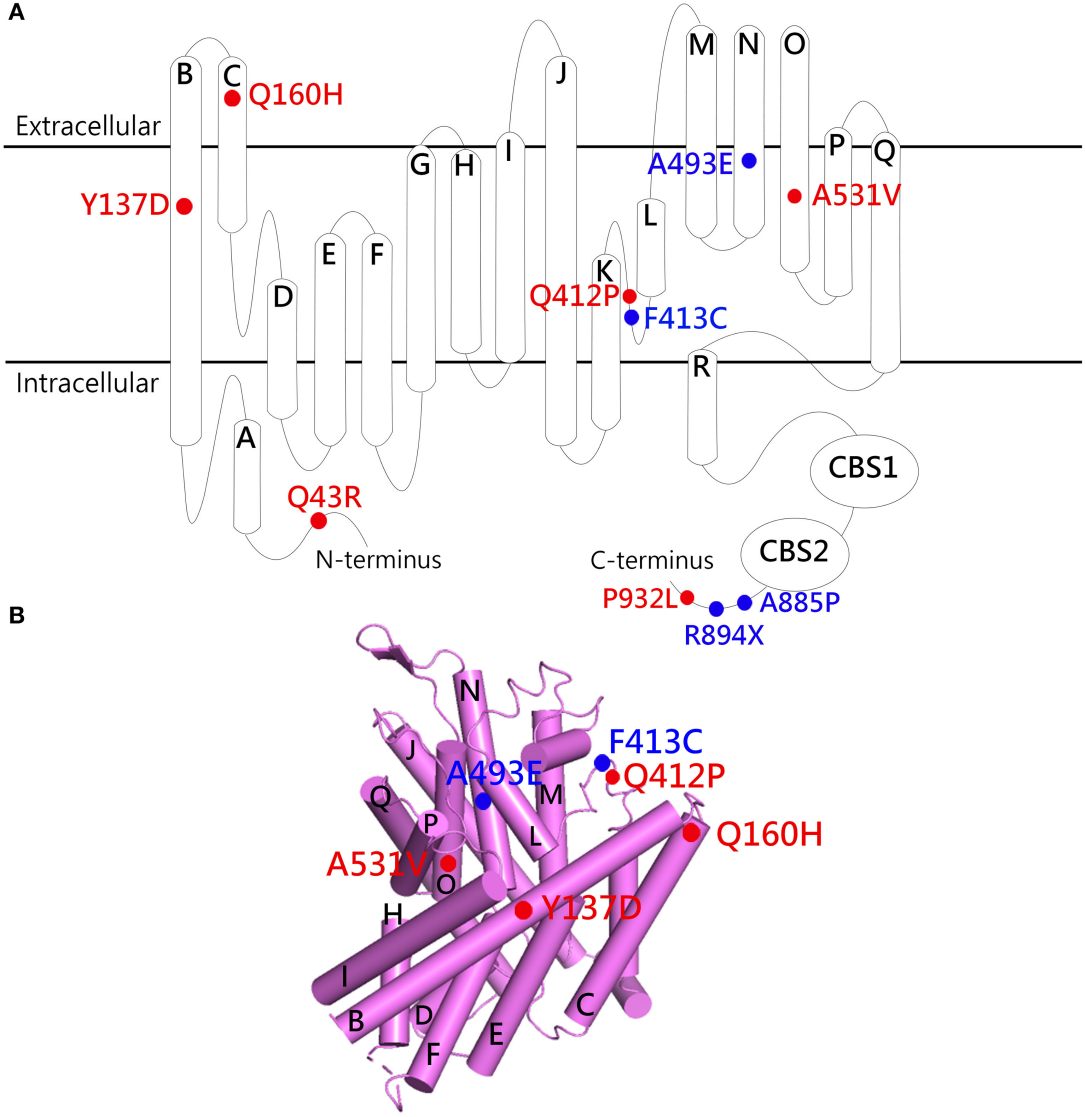
Structural localization of myotonia congenita-associated ClC-1 mutations with defective proteostasis. **(A)** Membrane topology of the ClC-1 subunit. **(B)** Lateral view of the transmembrane portion of the human ClC-1 cryo-EM structure (PDB code: 6QVC; presented using Pymol). The α-helices are shown as cylinders. Red labels, mutants with gating properties similar to those of WT; blue labels, mutants with altered gating properties. See Table 1 for more details.

To date, at least 10 myotonia-related ClC-1 mutants have been shown to display reduced protein expression at the plasma membrane (Table 1). Most of these mutations belong to recessive myotonia, with some others involving dominant or dual inheritance patterns. The locations of the mutations scatter over cytoplasmic N- and C-terminal regions, as well as transmembrane domains (Figure 3). Apart from proteostasis impairment, these ClC-1 mutants also show aberrant channel gating function (Figure 3 and Table 1). Given that the majority of previous studies of disease-causing mutations focus on functional characterizations without thorough biochemical analyses, it is conceivable that a significant fraction of the other known ClC-1 mutant channels with loss-of-function phenotypes may also be associated with defective proteostasis.

As far as proteostasis mechanisms are concerned, the most comprehensive analyses were performed for the A531V mutant (located at helix O), a recessively inherited mutation found prevalently in northern Finland and Scandinavia (12, 13). Despite an overall Po-V curve indistinguishable from that of the WT, the A531V mutant is associated with substantially reduced whole-cell current density (108, 109). Upon over-expression in both muscles and non-muscle cell lines, the A531V mutant exhibits significantly reduced protein levels that can be attributed to enhanced protein degradation (106, 108). Further studies show that the nature of this excessively reduced protein expression involves both proteasomal and lysosomal degradation, suggesting that the A531V mutant is associated with enhanced ERAD, as well as defective protein stability at the plasma membrane (108, 110, 111). Moreover, immunofluorescence analyses reveal a notable ER-retention pattern, indicating that the proteostasis defect of the A531V mutant also entails impaired membrane trafficking (106, 108). Together, these observations are consistent with the idea that the A531V mutant contains a serious folding anomaly that renders most of the mutant proteins undesirable for the quality control systems at the ER, Golgi, and plasma membrane, shifting ClC-1 proteostasis toward the degradation pathway.

Nonetheless, it remains unclear why a conservative alanine-to-valine mutation at residue 531 in the transmembrane helix O results in such a dramatic impairment in human ClC-1 proteostasis, and how the mutation subtly disrupts the structure of ClC-1 without notably affecting its biophysical properties. One possibility is that the misfolded ClC-1 mutant protein is predominantly misrouted in its proteostasis pathway, reducing the likelihood of correct folding; for the small fraction of mutant proteins passing the quality control system, the native protein conformation may be reasonably safeguarded, sparing the gating function of the channel. Another plausible idea is that the mutation may introduce an ER-retention signal or disrupt or an ER-export signal. Some of the known ER-retention or ER-export signal sequences in other ion channels and membrane proteins include RXR, KKXX, and VXXSL (137–140), none of which is present in residues 511–551 of the ClC-1 WT or the A531V mutant. Moreover, all known ER-retention/export signals are located in the intracellular region, whereas A531 is at the transmembrane helix O, adjacent to the dimer interface helices P and Q (Figure 3).

Although the evidence is as of yet not available, it is likely that some myotonia congenita-related ClC-1 mutations may result in aberrant membrane targeting/subcellular localization in skeletal muscles. One major limitation to better understanding of this critical question is that proteostasis pathways as well as subcellular localization patterns of ClC-1 channels *in situ* remain elusive. As discussed above in the “Structure and Function” section, it is still controversial whether the ClC-1 channel is located at the sarcolemma and/or the transverse-tubule system of skeletal muscles. Although biophysical and pharmacological studies support the presence of ClC-1-like Cl^−^ channel conductance in the transverse-tubules of rat skeletal muscles (62, 63, 72), immunohistochemical characterizations of muscle cryosections suggest that, in WT mice, the ClC-1 immunoreactivity is primarily found in the sarcolemmal membrane but not in the transverse-tubules of skeletal muscles (66). A similar sarcolemma-restricted immunohistochemical staining pattern is also observed in skeletal muscles of the arrested development of righting response (ADR) mouse (65, 141), a commonly used mouse model for recessive myotonia (82, 142). Nevertheless, the prominent sarcolemmal localization of ClC-1 in skeletal muscles seems to disappear immediately after the myofibers are isolated and maintained in cell culture conditions, suggesting that the subcellular localization of ClC-1 is tightly regulated by the physiological conditions within skeletal muscles (65). The mechanism underlying the foregoing discrepancy between physiological and immunological localizations of ClC-1 in skeletal muscles remains to be determined. This discrepancy may reflect the presence of certain ClC-1 splice variants in the transverse-tubule system that lack the proper epitopes for the antibodies used in the immunohistochemical studies (143), or the disruption of antibody-epitope interaction by endogenous ClC-1-binding proteins under certain physiological conditions.

## Proteostasis Network of Human ClC-1 Channel

As mentioned above, most of the newly synthesized, myotonia-causing A531V mutant proteins are incapable of passing the scrutiny of the cellular protein triage system and hence are subject to excessive proteasomal and lysosomal degradations. Even though application of the proteasome inhibitor MG132 effectively rescues the total protein level of the mutant ClC-1 channel, most of the MG132-rescued A531V proteins fail to be delivered to the plasma membrane (108). Accordingly, MG132 treatment does not rescue the reduced functional current of the mutant channel (108). Similarly, blocking the endosomal-lysosomal degradation system leads to a notable enhancement of A531V protein level, but fails to discernibly increase the whole-cell current density of the mutant channel (108). Together these results indicate that the defective surface protein density and the functional expression of the A531V mutant cannot be fixed by simply suppressing the degradation pathway. Rather, we must correct the impaired proteostasis of the mutant ClC-1 channel.

At the cellular level, proteostasis is maintained by over 2,000 macromolecules comprising chaperones/cochaperones, folding enzymes, and degradation and trafficking components, collectively known as the proteostasis network (130, 144). Until recently, the proteostasis network of human ClC-1 was virtually unknown. Nor was it clear how the ER and peripheral quality control systems recognize and mediate the degradation of disease-associated mutant ClC-1 proteins such as A531V.

In ERAD, which involves modification of misfolded proteins by the ubiquitin-proteasome system (Figure 2A), protein ubiquitination is mediated by a concerted action of multiple cytosolic and/or ER-resident enzymes, and may take place while transmembrane proteins are still located at the ER (128, 129, 145, 146). One of the key enzymes mediating protein ubiquitination is E3 ubiquitin ligase, which catalyzes the covalent linkage of ubiquitin to a substrate protein (145, 147). In higher eukaryotes, there are over 1000 distinct E3 ligases, divided into two major families: the homologous to E6-AP C-terminus (HECT) family and the really interesting new gene (RING) family (129, 148, 149). To date, over 20 HECT proteins and more than 600 RING proteins are known to express in human cells. We have demonstrated that polyubiquitination and degradation of human ClC-1 channel are catalyzed by two subtypes of the cullin (CUL)-RING E3 ubiquitin ligase complex, CUL4A/B-damage-specific DNA binding protein 1 (DDB1)-cereblon (CRBN) (110). CUL4A and 4B serve as scaffold proteins, facilitating the transfer of ubiquitin from the E2 ubiquitin-conjugating enzyme to a substrate protein, DDB1 is the adapter protein linking CUL4A/B and the substrate receptor, and CRBN works as the substrate receptor protein that directly recruits ClC-1 (150–152). This is the first direct evidence indicating that the CUL4 E3 ubiquitin ligase promotes degradation of ion channels. Incidentally, CUL E3 ligase activity is known to play an essential role in skeletal muscle homeostasis, myoblast differentiation, and myogenic differentiation of skeletal muscle stem cells (153, 154).

A cardinal process during protein biogenesis at the ER is the conformation surveillance of nascent polypeptides by chaperones and cochaperones that facilitate protein folding and thus minimize degradation/aggregation of non-native-state proteins (118, 155, 156). Moreover, for misfolded proteins that lose their stable conformations, chaperones/cochaperones assist them to the proteolytic pathway. We have also identified some of the key macromolecules participating in the protein quality control of human ClC-1 at the ER, including the interconnected molecular chaperones heat shock cognate protein 70 (Hsc70) and heat shock protein 90β (Hsp90β), and the cochaperones FK506-binding protein 8 (FKBP8 or FKBP38), activator of Hsp90 ATPase homolog 1 (Aha1), and Hsp70/Hsp90 organizing protein (HOP) (111). Hsc70 and Hsp90β are the constitutively active isoforms of Hsp70 and Hsp90, respectively, and both have been shown to take part in the ER quality control (155). FKBP8, Aha1, and HOP are well-established cochaperones for Hsp70 and Hsp90. The ER-resident membrane-anchored immunophilin FKBP8 may serve as a potential peptidyl-prolyl *cis-trans* isomerase, and the cytosolic proteins Aha1 and HOP regulate the ATPase activity of Hsp90 as well as the interaction of Hsp70 and Hsp90 (155, 157–159). All of the identified chaperones and cochaperones facilitate ClC-1 protein expression, and FKBP8 displays additional effect on promoting protein stability and membrane trafficking. Interestingly, we also noticed that Hsp90β and FKBP8 co-exist in the same protein complex with the E3 ligase scaffold protein CUL4, and appear to contribute to the regulation of CUL4 protein stability as well.

**Figure 4:**
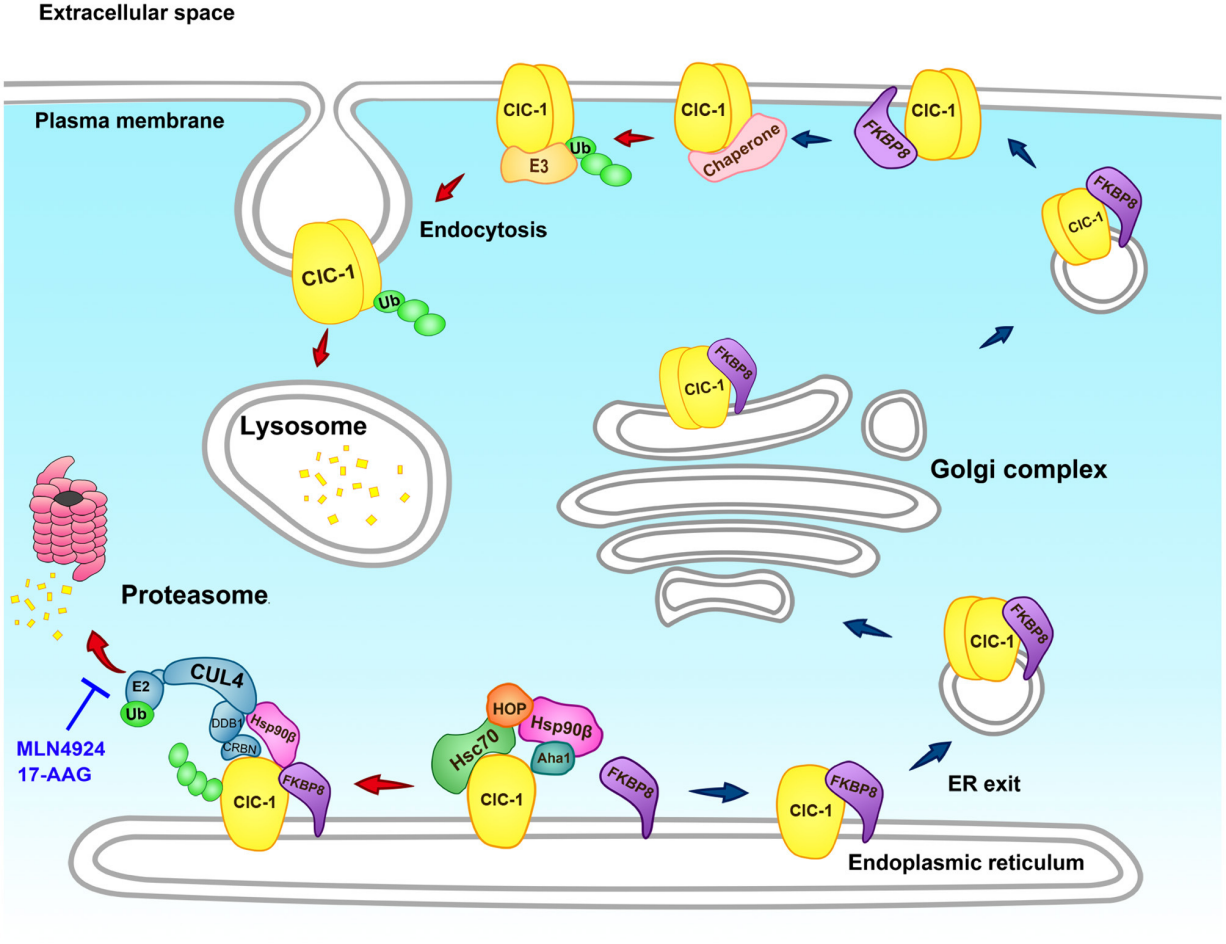
Schematic model of the proteostasis network of the human ClC-1 channel. The endoplasmic reticulum (ER) quality control system of the CLC-1 protein comprises of the constitutively expressed molecular chaperones Hsc70 and Hsp90β, as well as the cochaperones HOP, Aha1, and FKBP8. Hsc70 and HOP assist the early stage of ClC-1 folding, whereas Aha1, Hsp90β, and FKBP8 promote the late stage of ClC-1 folding. ER-associated degradation of ClC-1 is mediated by the CUL4-DDB1-CRBN E3 ubiquitin ligase complex that catalyzes the transfer of ubiquitin (Ub) from the E2 ubiquitin-conjugating enzyme (E2) for covalent linkage to ClC-1. Ubiquitinated ClC-1 is targeted for eventual degradation by the proteasome. Hsp90β and FKBP8 may additionally regulate ER-associated degradation of ClC-1 by modulating the protein stability of CRBN, the substrate receptor of the CUL4-DDB1-CRBN ligase complex. Proteasomal degradation of ClC-1 can be effectively attenuated by the cullin E3 ligase blocker MLN4924 and the Hsp90 inhibitor 17-AAG. Moreover, FKBP8 is essential for ER exit and membrane trafficking of ClC-1. At the plasma membrane, FKBP8 further promotes surface ClC-1 protein stability. Other chaperones/cochaperones may also contribute to the peripheral quality control system of ClC-1. Misfolded ClC-1 is subject to ubiquitination by the as yet unknown E3 ubiquitin ligase (E3), followed by endocytosis and lysosomal degradation.

Figure 4 outlines our current model on the proteostasis network of human ClC-1 channel. Hsc70 and HOP may facilitate the early protein biogenesis process of ClC-1, followed by a concerted action by Aha1, Hsp90β, and FKBP8 (the Hsp90β cycle) to further promote ClC-1 folding. Hsp90β and FKBP8 may also regulate the degradation of misfolded ClC-1 by the CUL4-DDB1-CRBN E3 ligase complex. We propose that, in the ER quality control, Hsp90β may serve as a molecular hub assisting the interaction of ClC-1 with Aha1, FKBP8, and CUL4, and therefore dynamically couple the ClC-1 protein folding and degradation pathways.

Our recent biochemical analyses suggest that, outside the ER, FKBP8 co-localizes with ClC-1 at both the Golgi complex and the plasma membrane; moreover, at the cell surface, FKBP8 enhances membrane ClC-1 protein level and promotes surface ClC-1 stability (160). Therefore, as depicted in Figure 4, we further propose that FKBP8 contributes to the ER export, membrane trafficking, and peripheral quality control of the human ClC-1 channel. It is an open question whether the rest of the chaperones/cochaperones implicated in ClC-1 ER quality control also play a role in the proteostasis of this Cl^−^ channel at the cell surface. In addition, the molecular nature of the E3 ligase catalyzing cell surface ClC-1 ubiquitination and the ensuing endosomal-lysosomal degradation mechanism is still unclear.

## Clinical Significance

Current treatment for myotonia congenita primarily involves reduction of muscle tone by suppressing action potential firing in skeletal muscles. The medications prescribed for treating non-dystrophic myotonia include the anti-arrhythmic agent mexiletine and the anti-epileptic agent lamotrigine (161–163). Both drugs effectively block voltage-gated Na^+^ channels and repetitive action potential firing in a use-dependent manner (164–167). At present, there is no treatment specifically designed to correct defective gating or proteostasis of disease-causing mutant ClC-1 channels.

In direct contrast to the aforementioned lack of effect of proteasomal/lysosomal inhibitors on enhancing functional current (108), suppression of CUL4A/B E3 ligase and promotion of chaperone/cochaperone activities significantly enhance the surface protein level and whole-cell current density of the myotonia-causing A531V mutant (110, 111). The results thus suggest that direct manipulation of the proteostasis network effectively corrects the impaired biogenesis of misfolded ClC-1 protein. Importantly, we identified two emerging small-molecule anti-cancer agents that may ameliorate defective proteostasis of ClC-1: MLN4924 and 17-allylamino-17-demethoxygeldanamycin (17-AAG) [Figure 4; (110, 111)]. MLN4924, which inhibits cullin E3 ubiquitin ligase activity by blocking the conjugation of the ubiquitin-like molecule NEDD8 to the cullin scaffold protein (168, 169), is currently undergoing clinical trials in cancer patients (170–173). The molecule 17-AAG, which suppresses the ATPase activity of Hsp90 by blocking ATP binding to the chaperone (174, 175), is also being tested in various clinical trials as an anti-cancer agent (174–176).

For human diseases caused by proteostasis impairment, it is essential to identify or develop novel biological and chemical therapeutics aiming at optimizing protein conformation and enhancing proteostasis capacity (130, 177, 178). For example, the Hsp90 inhibitor 17-AAG may serve as a potential pharmacological chaperone (pharmacochaperone) for modifying impaired proteostasis network of neurodegenerative diseases such as motor neuron degeneration and spinocerebellar ataxia (131, 179, 180). Therefore, our demonstration that 17-AAG improves the defective proteostasis of A531V raises a possibility that 17-AAG and other small-molecule pharmacochaperones could be clinically applied in the future to correct the protein folding defect of myotonia-causing ClC-1 mutant proteins.

The clinical implication of correcting defective ClC-1 proteostasis with pharmacological proteostasis network modifiers is actually beyond the scope of myotonia congenita, as ClC-1 dysfunction has been identified in other pathological conditions associated with anomalous skeletal muscle function. In myotonic dystrophy type 1 and 2 (DM1 and DM2), for example, mutations in the *DMPK* and *ZNF9*/*CNBP* genes, respectively, disrupt the alternative splicing of the *CLCN1* gene, creating a secondary reduction in sarcolemmal ClC-1 protein expression and current density (181–184). Correction of ClC-1 splicing with an antisense-induced exon skipping technique appears to eliminate the myotonia phenotype in a mouse model of DM1 (185). Interestingly, several studies further indicate the presence of significant co-segregation of DM2 with myotonia congenita-causing ClC-1 mutations such as F413C and R894X, both associated with defective ClC-1 proteostasis [Figure 3 and Table 1; (186, 187)]. Similar to the pathological mechanism of myotonic dystrophy, emerging evidence suggests that Huntington disease also involves aberrant mRNA splicing of the *CLCN1* gene, thereby manifesting as hyperexcitability of skeletal muscles (188, 189). Moreover, statins, among the most effective agents in treating dyslipidemia, are associated with a significant incidence of myotoxicity (manifesting as symptoms such as muscle weakness, muscle pain, muscle stiffness, and muscle cramps), and may instigate considerably reduced ClC-1 protein expression and Cl^−^ conductance in skeletal muscles (190–193). Significantly, despite the possibility that statins may cause notable Ca^2+^ release from mitochondria and sarcoplasmic reticulum, statin-induced down-regulation of ClC-1 expression in skeletal muscles cannot be explained by reduced *CLCN1* transcription or enhanced PKC-mediated inhibition of ClC-1 channel activation (191, 192), suggesting the potential presence of a statin-induced disruption of ClC-1 proteostasis. Therefore, future development of specific and effective ClC-1 proteostasis modifiers may shed light on new therapeutic strategies for ameliorating the foregoing debilitating muscle symptoms.

Another issue of clinical relevancy concerns CRBN, the ClC-1-binding substrate receptor protein of the CUL4 E3 ligase complex. CRBN is known to be the binding target of thalidomide and lenalidomide (194–196), both immunomodulatory drugs used for the treatment of multiple myeloma (197, 198). Common side effects of thalidomide and lenalidomide treatments include muscle weakness and muscle cramps (197, 199), suggesting the presence of drug-induced hyperexcitability in skeletal muscles. Importantly, both thalidomide and lenalidomide suppress CUL4-DDB1-mediated ubiquitination and degradation of CRBN, thereby effectively promoting the degradation of some substrate proteins for the CUL4-DDB1-CRBN E3 ubiquitin ligase complex (200, 201). Given our previous demonstration that CUL4-DDB1-CRBN mediates ERAD of human ClC-1 channel and that over-expression of CRBN significantly suppresses ClC-1 protein level (110), it is possible that thalidomide/lenalidomide-induced muscle cramps observed in myeloma patients is in part attributable to enhanced degradation of human ClC-1 channel in skeletal muscles. In light of our proof-of-concept evidence that the small-molecule CUL4 inhibitor MLN4924 can effectively promote surface expression and current density of ClC-1 (110), relief from thalidomide/lenalidomide-induced side effects in skeletal muscles may be achievable in the future by developing muscle-specific, MLN4924-like CUL4-DDB1-CRBN E3 ligase modulators.

As elaborated in the “Structure and Function” section, depending on muscle fiber types, regulation of skeletal muscle fatigue may involve reduced and enhanced activation of ClC-1 channel through PKC activation and ATP diminishment, respectively. A recent study on the effect of exercise training on skeletal muscles in human subjects further suggests that ClC-1 protein abundance is higher in the fast-twitch than in the slow-twitch muscle fibers, and that, compared to recreationally active individuals, trained cyclists are associated with lower ClC-1 protein abundance (202). These observations imply that low ClC-1 abundance enhances muscle excitability and contractility and is beneficial for exercise performance. Although the role of transcriptional regulation of ClC-1 expression in skeletal muscles is well documented (20), it remains an open question whether cellular maintenance of proteostasis may also contribute to developmental and physiological controls of ClC-1 protein abundance. Most importantly, the foregoing results appear to suggest an intriguing ClC-1 proteostasis adaptation mechanism that accommodates the differential physiological roles of fast- and slow-twitch fibers, and improves muscle contraction efficiency in response to exercise training. It is therefore imperative to understand the detailed proteostasis network of ClC-1 for elucidating the physiology of muscle training and the pathophysiology of muscle disorders.

## Conclusion

Myotonia congenita is a ClC-1 channelopathy that involves skeletal muscle hyperexcitability due to a significant loss of muscle Cl^−^ conductance. Comprehensive genetic analyses have identified over 200 mutations in the human *CLCN1* gene associated with this hereditary disease. Biophysical investigations in the last three decades have revealed the mechanistic roles of aberrant gating and permeation properties in various myotonia-causing ClC-1 mutants. Determination of the cryo-EM structure of human ClC-1 provides further insight to the structural-functional mechanisms underlying dominant and recessive forms of myotonia congenita. Overwhelming evidence, however, indicates that aberrant channel gating and permeation *per se* are insufficient to explain the molecular pathophysiology of myotonia congenita, which can also result from abnormal biochemical and cell biological properties of ClC-1. Therefore, the field is in need of advanced understanding of theses aspects such as *in vivo* subcellular localization patterns and post-translational regulations. Another crucial task concerns the illumination of specific proteostasis mechanisms governing the biogenesis, trafficking, and quality control of WT and misfolded mutant ClC-1 proteins. Detailed elucidation of the ClC-1 proteostasis network may hold great promise for identifying ClC-1-specific abnormalities that may serve as targets for novel pharmacological interventions of myotonia congenita, as well as other pathological conditions causing skeletal muscle dysfunctions.

## Author Contributions

S-JF, C-YY, Y-JP, and C-TH: preparation and revision of table and figures. C-JJ, T-YC, and C-YT: writing and revision of manuscript.

### Conflict of Interest

The authors declare that the research was conducted in the absence of any commercial or financial relationships that could be construed as a potential conflict of interest.

## References

[1] Meola G Cardani R. Myotonic dystrophies: an update on clinical aspects, genetic, pathology, and molecular pathomechanisms. Biochim Biophys Acta. (2015) 1852:594–606. 10.1016/j.bbadis.2014.05.019 24882752

[2] Udd B Krahe R. The myotonic dystrophies: molecular, clinical, and therapeutic challenges. Lancet Neurol. (2012) 11:891–905. 10.1016/S1474-4422(12)70204-1 22995693

[3] Wenninger S Montagnese F Schoser B. Core clinical phenotypes in myotonic dystrophies. Front Neurol. (2018) 9:303. 10.3389/fneur.2018.00303 29770119 PMC5941986

[4] Raja Rayan DL Hanna MG. Skeletal muscle channelopathies: nondystrophic myotonias and periodic paralysis. Curr Opin Neurol. (2010) 23:466–76. 10.1097/WCO.0b013e32833cc97e 20634695

[5] Cannon SC. Pathomechanisms in channelopathies of skeletal muscle and brain. Annu Rev Neurosci. (2006) 29:387–415. 10.1146/annurev.neuro.29.051605.112815 16776591

[6] Suetterlin K Mannikko R Hanna MG. Muscle channelopathies: recent advances in genetics, pathophysiology and therapy. Curr Opin Neurol. (2014) 27:583–90. 10.1097/WCO.0000000000000127 25188014

[7] Kullmann DM. Neurological channelopathies. Annu Rev Neurosci. (2010) 33:151–72. 10.1146/annurev-neuro-060909-153122 20331364

[8] Koch MC Steinmeyer K Lorenz C Ricker K Wolf F Otto M. The skeletal muscle chloride channel in dominant and recessive human myotonia. Science. (1992) 257:797–800. 10.1126/science.1379744 1379744

[9] Lossin C George AL Jr. Myotonia congenita. Adv Genet. (2008) 63:25–55. 10.1016/S0065-2660(08)01002-X 19185184

[10] Pusch M. Myotonia caused by mutations in the muscle chloride channel gene CLCN1. Hum Mutat. (2002) 19:423–34. 10.1002/humu.10063 11933197

[11] Jurkat-Rott K Lerche H Lehmann-Horn F. Skeletal muscle channelopathies. J Neurol. (2002) 249:1493–502. 10.1007/s00415-002-0871-5 12420087

[12] Papponen H Toppinen T Baumann P Myllyla V Leisti J Kuivaniemi H. Founder mutations and the high prevalence of myotonia congenita in northern Finland. Neurology. (1999) 53:297–302. 10.1212/WNL.53.2.297 10430417

[13] Sun C Tranebjaerg L Torbergsen T Holmgren G Van Ghelue M. Spectrum of CLCN1 mutations in patients with myotonia congenita in Northern Scandinavia. Eur J Hum Genet. (2001) 9:903–9. 10.1038/sj.ejhg.5200736 11840191

[14] Emery AE. Population frequencies of inherited neuromuscular diseases–a world survey. Neuromuscul Disord. (1991) 1:19–29. 10.1016/0960-8966(91)90039-U 1822774

[15] Portaro S Altamura C Licata N Camerino GM Imbrici P Musumeci O. Clinical, molecular, and functional characterization of CLCN1 mutations in three families with recessive myotonia congenita. Neuromolecular Med. (2015) 17:285–96. 10.1007/s12017-015-8356-8 26007199 PMC4534513

[16] Chen TY. Structure and function of clc channels. Annu Rev Physiol. (2005) 67:809–39. 10.1146/annurev.physiol.67.032003.153012 15709979

[17] Jentsch TJ Poet M Fuhrmann JC Zdebik AA. Physiological functions of CLC Cl- channels gleaned from human genetic disease and mouse models. Annu Rev Physiol. (2005) 67:779–807. 10.1146/annurev.physiol.67.032003.153245 15709978

[18] Picollo A Pusch M. Chloride/proton antiporter activity of mammalian CLC proteins ClC-4 and ClC-5. Nature. (2005) 436:420–3. 10.1038/nature03720 16034421

[19] Scheel O Zdebik AA Lourdel S Jentsch TJ. Voltage-dependent electrogenic chloride/proton exchange by endosomal CLC proteins. Nature. (2005) 436:424–7. 10.1038/nature03860 16034422

[20] Jentsch TJ Pusch M. CLC chloride channels and transporters: structure, function, physiology, and disease. Physiol Rev. (2018) 98:1493–590. 10.1152/physrev.00047.2017 29845874

[21] Feng L Campbell EB Hsiung Y MacKinnon R. Structure of a eukaryotic CLC transporter defines an intermediate state in the transport cycle. Science. (2010) 330:635–41. 10.1126/science.1195230 20929736 PMC3079386

[22] Dutzler R Campbell EB Cadene M Chait BT MacKinnon R. X-ray structure of a ClC chloride channel at 3.0 A reveals the molecular basis of anion selectivity. Nature. (2002) 415:287–94. 10.1038/415287a 11796999

[23] Dutzler R Campbell EB MacKinnon R. Gating the selectivity filter in ClC chloride channels. Science. (2003) 300:108–12. 10.1126/science.1082708 12649487

[24] Wang K Preisler SS Zhang L Cui Y Missel JW Gronberg C. Structure of the human ClC-1 chloride channel. PLoS Biol. (2019) 17:e3000218. 10.1371/journal.pbio.3000218 31022181 PMC6483157

[25] Park E MacKinnon R. Structure of the CLC-1 chloride channel from Homo sapiens. Elife. (2018) 7:e36629. 10.7554/eLife.36629 29809153 PMC6019066

[26] Park E Campbell EB MacKinnon R. Structure of a CLC chloride ion channel by cryo-electron microscopy. Nature. (2017) 541:500–5. 10.1038/nature20812 28002411 PMC5576512

[27] Tseng PY Yu WP Liu HY Zhang XD Zou X Chen TY. Binding of ATP to the CBS domains in the C-terminal region of CLC-1. J Gen Physiol. (2011) 137:357–68. 10.1085/jgp.201010495 21444658 PMC3068280

[28] Miller C. Open-state substructure of single chloride channels from Torpedo electroplax. Philos Trans R Soc Lond B Biol Sci. (1982) 299:401–11. 10.1098/rstb.1982.0140 6130538

[29] Miller C White MM. Dimeric structure of single chloride channels from Torpedo electroplax. Proc Natl Acad Sci USA. (1984) 81:2772–5. 10.1073/pnas.81.9.2772 6326143 PMC345152

[30] Saviane C Conti F Pusch M. The muscle chloride channel ClC-1 has a double-barreled appearance that is differentially affected in dominant and recessive myotonia. J Gen Physiol. (1999) 113:457–68. 10.1085/jgp.113.3.457 10051520 PMC2222904

[31] Fahlke C Knittle T Gurnett CA Campbell KP George AL Jr. Subunit stoichiometry of human muscle chloride channels. J Gen Physiol. (1997) 109:93–104. 10.1085/jgp.109.1.93 8997668 PMC2217051

[32] Weinreich F Jentsch TJ. Pores formed by single subunits in mixed dimers of different CLC chloride channels. J Biol Chem. (2001) 276:2347–53. 10.1074/jbc.M005733200 11035003

[33] Accardi A Pusch M. Fast and slow gating relaxations in the muscle chloride channel CLC-1. J Gen Physiol. (2000) 116:433–44. 10.1085/jgp.116.3.433 10962018 PMC2233683

[34] Rychkov GY Pusch M Astill DS Roberts ML Jentsch TJ Bretag AH. Concentration and pH dependence of skeletal muscle chloride channel ClC-1. J Physiol. (1996) 497(Pt 2):423–35. 10.1113/jphysiol.1996.sp021778 8961185 PMC1160994

[35] Hebeisen S Fahlke C. Carboxy-terminal truncations modify the outer pore vestibule of muscle chloride channels. Biophys J. (2005) 89:1710–20. 10.1529/biophysj.104.056093 15980168 PMC1366675

[36] Chen MF Chen TY. Different fast-gate regulation by external Cl(-) and H(+) of the muscle-type ClC chloride channels. J Gen Physiol. (2001) 118:23–32. 10.1085/jgp.118.1.23 11429442 PMC2233746

[37] Chen TY Miller C. Nonequilibrium gating and voltage dependence of the ClC-0 Cl- channel. J Gen Physiol. (1996) 108:237–50. 10.1085/jgp.108.4.237 8894974 PMC2229332

[38] Pusch M Steinmeyer K Koch MC Jentsch TJ. Mutations in dominant human myotonia congenita drastically alter the voltage dependence of the CIC-1 chloride channel. Neuron. (1995) 15:1455–63. 10.1016/0896-6273(95)90023-3 8845168

[39] Dutzler R. The structural basis of ClC chloride channel function. Trends Neurosci. (2004) 27:315–20. 10.1016/j.tins.2004.04.001 15165735

[40] Dutzler R. Structural basis for ion conduction and gating in ClC chloride channels. FEBS Lett. (2004) 564:229–33. 10.1016/S0014-5793(04)00210-8 15111101

[41] Chen TY. Coupling gating with ion permeation in ClC channels. Sci STKE. (2003) 2003:pe23. 10.1126/stke.2003.188.pe23 12824475

[42] Duffield M Rychkov G Bretag A Roberts M. Involvement of helices at the dimer interface in ClC-1 common gating. J Gen Physiol. (2003) 121:149–61. 10.1085/jgp.20028741 12566541 PMC2217322

[43] Stolting G Fischer M Fahlke C. ClC-1 and ClC-2 form hetero-dimeric channels with novel protopore functions. Pflugers Arch. (2014) 466:2191–204. 10.1007/s00424-014-1490-6 24638271

[44] Accardi A Ferrera L Pusch M. Drastic reduction of the slow gate of human muscle chloride channel (ClC-1) by mutation C277S. J Physiol. (2001) 534:745–52. 10.1111/j.1469-7793.2001.00745.x 11483705 PMC2278749

[45] Weinberger S Wojciechowski D Sternberg D Lehmann-Horn F Jurkat-Rott K Becher T. Disease-causing mutations C277R and C277Y modify gating of human ClC-1 chloride channels in myotonia congenita. J Physiol. (2012) 590:3449–64. 10.1113/jphysiol.2012.232785 22641783 PMC3547262

[46] Cederholm JM Rychkov GY Bagley CJ Bretag AH. Inter-subunit communication and fast gate integrity are important for common gating in hClC-1. Int J Biochem Cell Biol. (2010) 42:1182–8. 10.1016/j.biocel.2010.04.004 20398785

[47] Bykova EA Zhang XD Chen TY Zheng J. Large movement in the C terminus of CLC-0 chloride channel during slow gating. Nat Struct Mol Biol. (2006) 13:1115–9. 10.1038/nsmb1176 17115052

[48] Zhang XD Tseng PY Chen TY. ATP inhibition of CLC-1 is controlled by oxidation and reduction. J Gen Physiol. (2008) 132:421–8. 10.1085/jgp.200810023 18824589 PMC2553389

[49] Tseng PY Bennetts B Chen TY. Cytoplasmic ATP inhibition of CLC-1 is enhanced by low pH. J Gen Physiol. (2007) 130:217–21. 10.1085/jgp.200709817 17664348 PMC2151639

[50] Bennetts B Yu Y Chen TY Parker MW. Intracellular beta-nicotinamide adenine dinucleotide inhibits the skeletal muscle ClC-1 chloride channel. J Biol Chem. (2012) 287:25808–20. 10.1074/jbc.M111.327551 22689570 PMC3406667

[51] Bennetts B Parker MW. Molecular determinants of common gating of a ClC chloride channel. Nat Commun. (2013) 4:2507. 10.1038/ncomms3507 24064982

[52] Steinmeyer K Ortland C Jentsch TJ. Primary structure and functional expression of a developmentally regulated skeletal muscle chloride channel. Nature. (1991) 354:301–4. 10.1038/354301a0 1659664

[53] Zhang XD Morishima S Ando-Akatsuka Y Takahashi N Nabekura T Inoue H. Expression of novel isoforms of the CIC-1 chloride channel in astrocytic glial cells *in vitro*. Glia. (2004) 47:46–57. 10.1002/glia.20024 15139012

[54] Jentsch TJ Maritzen T Zdebik AA Chloride channel diseases resulting from impaired transepithelial transport or vesicular function. J Clin Invest. (2005) 115:2039–46. 10.1172/JCI25470 16075045 PMC1180548

[55] Jentsch TJ Stein V Weinreich F Zdebik AA. Molecular structure and physiological function of chloride channels. Physiol Rev. (2002) 82:503–68. 10.1152/physrev.00029.2001 11917096

[56] Pedersen TH Riisager A de Paoli FV Chen TY Nielsen OB. Role of physiological ClC-1 Cl- ion channel regulation for the excitability and function of working skeletal muscle. J Gen Physiol. (2016) 147:291–308. 10.1085/jgp.201611582 27022190 PMC4810071

[57] Tang CY Chen TY. Physiology and pathophysiology of CLC-1: mechanisms of a chloride channel disease, myotonia. J Biomed Biotechnol. (2011) 2011:685328. 10.1155/2011/685328 22187529 PMC3237021

[58] Bryant SH Morales-Aguilera A. Chloride conductance in normal and myotonic muscle fibres and the action of monocarboxylic aromatic acids. J Physiol. (1971) 219:367–83. 10.1113/jphysiol.1971.sp009667 5316641 PMC1331636

[59] Dulhunty AF. Distribution of potassium and chloride permeability over the surface and T-tubule membranes of mammalian skeletal muscle. J Membr Biol. (1979) 45:293–310. 10.1007/BF01869290 458844

[60] Bretag AH. Muscle chloride channels. Physiol Rev. (1987) 67:618–724. 10.1152/physrev.1987.67.2.618 2436244

[61] Adrian RH Bryant SH. On the repetitive discharge in myotonic muscle fibres. J Physiol. (1974) 240:505–15. 10.1113/jphysiol.1974.sp010620 4420758 PMC1331026

[62] Dutka TL Murphy RM Stephenson DG Lamb GD. Chloride conductance in the transverse tubular system of rat skeletal muscle fibres: importance in excitation-contraction coupling and fatigue. J Physiol. (2008) 586:875–87. 10.1113/jphysiol.2007.144667 18033812 PMC2375618

[63] Coonan JR Lamb GD. Effect of transverse-tubular chloride conductance on excitability in skinned skeletal muscle fibres of rat and toad. J Physiol. (1998) 509(Pt 2):551–64. 10.1111/j.1469-7793.1998.551bn.x 9575303 PMC2230972

[64] Palade PT Barchi RL. Characteristics of the chloride conductance in muscle fibers of the rat diaphragm. J Gen Physiol. (1977) 69:325–42. 10.1085/jgp.69.3.325 15046 PMC2215020

[65] Papponen H Kaisto T Myllyla VV Myllyla R Metsikko K. Regulated sarcolemmal localization of the muscle-specific ClC-1 chloride channel. Exp Neurol. (2005) 191:163–73. 10.1016/j.expneurol.2004.07.018 15589523

[66] Lueck JD Rossi AE Thornton CA Campbell KP Dirksen RT. Sarcolemmal-restricted localization of functional ClC-1 channels in mouse skeletal muscle. J Gen Physiol. (2010) 136:597–613. 10.1085/jgp.201010526 21078869 PMC2995150

[67] Zifarelli G Pusch M. Relaxing messages from the sarcolemma. J Gen Physiol. (2010) 136:593–6. 10.1085/jgp.201010567 21078870 PMC2995151

[68] Lamb GD Murphy RM Stephenson DG. On the localization of ClC-1 in skeletal muscle fibers. J Gen Physiol. (2011) 137:327–9; author reply 331–3. 10.1085/jgp.201010580 21357735 PMC3047610

[69] Fahlke C. Chloride channels take center stage in a muscular drama. J Gen Physiol. (2011) 137:17–9. 10.1085/jgp.201010574 21149545 PMC3010059

[70] DiFranco M Herrera A Vergara JL. Chloride currents from the transverse tubular system in adult mammalian skeletal muscle fibers. J Gen Physiol. (2011) 137:21–41. 10.1085/jgp.201010496 21149546 PMC3010054

[71] Cairns SP Lindinger MI. Do multiple ionic interactions contribute to skeletal muscle fatigue? J Physiol. (2008) 586:4039–54. 10.1113/jphysiol.2008.155424 18591187 PMC2652190

[72] Pedersen TH Nielsen OB Lamb GD Stephenson DG. Intracellular acidosis enhances the excitability of working muscle. Science. (2004) 305:1144–7. 10.1126/science.1101141 15326352

[73] Baekgaard Nielsen O de Paoli FV Riisager A Pedersen TH. Chloride channels take center stage in acute regulation of excitability in skeletal muscle: implications for fatigue. Physiology. (2017) 32:425–34. 10.1152/physiol.00006.2015 29021362

[74] Allen DG Lamb GD Westerblad H. Skeletal muscle fatigue: cellular mechanisms. Physiol Rev. (2008) 88:287–332. 10.1152/physrev.00015.2007 18195089

[75] Roos A Boron WF. Intracellular pH transients in rat diaphragm muscle measured with DMO. Am J Physiol. (1978) 235:C49–54. 10.1152/ajpcell.1978.235.1.C49 27989

[76] Wilson JR McCully KK Mancini DM Boden B Chance B. Relationship of muscular fatigue to pH and diprotonated Pi in humans: a 31P-NMR study. J Appl Physiol. (1988) 64:2333–9. 10.1152/jappl.1988.64.6.2333 3403417

[77] Riisager A de Paoli FV Yu WP Pedersen TH Chen TY Nielsen OB. Protein kinase C-dependent regulation of ClC-1 channels in active human muscle and its effect on fast and slow gating. J Physiol. (2016) 594:3391–406. 10.1113/JP271556 26857341 PMC4908021

[78] Bennetts B Parker MW Cromer BA. Inhibition of skeletal muscle ClC-1 chloride channels by low intracellular pH and ATP. J Biol Chem. (2007) 282:32780–91. 10.1074/jbc.M703259200 17693413

[79] Rosenbohm A Rudel R Fahlke C. Regulation of the human skeletal muscle chloride channel hClC-1 by protein kinase C. J Physiol. (1999) 514(Pt 3):677–85. 10.1111/j.1469-7793.1999.677ad.x 9882739 PMC2269114

[80] Karatzaferi C de Haan A Ferguson RA van Mechelen W Sargeant AJ. Phosphocreatine and ATP content in human single muscle fibres before and after maximum dynamic exercise. Pflugers Arch. (2001) 442:467–74. 10.1007/s004240100552 11484780

[81] Gronemeier M Condie A Prosser J Steinmeyer K Jentsch TJ Jockusch H. Nonsense and missense mutations in the muscular chloride channel gene Clc-1 of myotonic mice. J Biol Chem. (1994) 269:5963–7. 8119941

[82] Steinmeyer K Klocke R Ortland C Gronemeier M Jockusch H Grunder S Jentsch TJ. Inactivation of muscle chloride channel by transposon insertion in myotonic mice. Nature. (1991) 354:304–8. 10.1038/354304a0 1659665

[83] Rhodes TH Vite CH Giger U Patterson DF Fahlke C George AL Jr. A missense mutation in canine C1C-1 causes recessive myotonia congenita in the dog. FEBS Lett. (1999) 456:54–8. 10.1016/S0014-5793(99)00926-6 10452529

[84] Beck CL Fahlke C George AL Jr. Molecular basis for decreased muscle chloride conductance in the myotonic goat. Proc Natl Acad Sci USA. (1996) 93:11248–52. 10.1073/pnas.93.20.11248 8855341 PMC38315

[85] Adrian RH Marshall MW. Action potentials reconstructed in normal and myotonic muscle fibres. J Physiol. (1976) 258:125–43. 10.1113/jphysiol.1976.sp011410 940049 PMC1308963

[86] Lipicky RJ Bryant SH. Sodium, potassium, and chloride fluxes in intercostal muscle from normal goats and goats with hereditary myotonia. J Gen Physiol. (1966) 50:89–111. 10.1085/jgp.50.1.89 5971035 PMC2225635

[87] Colding-Jorgensen E. Phenotypic variability in myotonia congenita. Muscle Nerve. (2005) 32:19–34. 10.1002/mus.20295 15786415

[88] Fialho D Schorge S Pucovska U Davies NP Labrum R Haworth A. Chloride channel myotonia: exon 8 hot-spot for dominant-negative interactions. Brain. (2007) 130:3265–74. 10.1093/brain/awm248 17932099

[89] Fahlke C Rudel R Mitrovic N Zhou M George AL Jr. An aspartic acid residue important for voltage-dependent gating of human muscle chloride channels. Neuron. (1995) 15:463–72. 10.1016/0896-6273(95)90050-0 7646898

[90] Zhang J Sanguinetti MC Kwiecinski H Ptacek LJ. Mechanism of inverted activation of ClC-1 channels caused by a novel myotonia congenita mutation. J Biol Chem. (2000) 275:2999–3005. 10.1074/jbc.275.4.2999 10644771

[91] Ryan A Rudel R Kuchenbecker M Fahlke C. A novel alteration of muscle chloride channel gating in myotonia levior. J Physiol. (2002) 545:345–54. 10.1113/jphysiol.2002.027037 12456816 PMC2290694

[92] Ha K Kim SY Hong C Myeong J Shin JH Kim DS. Electrophysiological characteristics of six mutations in hClC-1 of Korean patients with myotonia congenita. Mol Cells. (2014) 37:202–12. 10.14348/molcells.2014.2267 24625573 PMC3969040

[93] Imbrici P Altamura C Pessia M Mantegazza R Desaphy JF Camerino DC. ClC-1 chloride channels: state-of-the-art research and future challenges. Front Cell Neurosci. (2015) 9:156. 10.3389/fncel.2015.00156 25964741 PMC4410605

[94] Wollnik B Kubisch C Steinmeyer K Pusch M. Identification of functionally important regions of the muscular chloride channel CIC-1 by analysis of recessive and dominant myotonic mutations. Hum Mol Genet. (1997) 6:805–11. 10.1093/hmg/6.5.805 9158157

[95] Kubisch C Schmidt-Rose T Fontaine B Bretag AH Jentsch TJ. ClC-1 chloride channel mutations in myotonia congenita: variable penetrance of mutations shifting the voltage dependence. Hum Mol Genet. (1998) 7:1753–60. 10.1093/hmg/7.11.1753 9736777

[96] Skalova D Zidkova J Vohanka S Mazanec R Musova Z Vondracek P. CLCN1 mutations in Czech patients with myotonia congenita, *in silico* analysis of novel and known mutations in the human dimeric skeletal muscle chloride channel. PLoS ONE. (2013) 8:e82549. 10.1371/journal.pone.0082549 24349310 PMC3859631

[97] Vindas-Smith R Fiore M Vasquez M Cuenca P Del Valle G Lagostena L. Identification and functional characterization of CLCN1 mutations found in nondystrophic myotonia patients. Hum Mutat. (2016) 37:74–83. 10.1002/humu.22916 26510092

[98] Ronstedt K Sternberg D Detro-Dassen S Gramkow T Begemann B Becher T. Impaired surface membrane insertion of homo- and heterodimeric human muscle chloride channels carrying amino-terminal myotonia-causing mutations. Sci Rep. (2015) 5:15382. 10.1038/srep15382 26502825 PMC4621517

[99] Simpson BJ Height TA Rychkov GY Nowak KJ Laing NG Hughes BP. Characterization of three myotonia-associated mutations of the CLCN1 chloride channel gene via heterologous expression. Hum Mutat. (2004) 24:185. 10.1002/humu.9260 15241802

[100] Zhang J Bendahhou S Sanguinetti MC Ptacek LJ. Functional consequences of chloride channel gene (CLCN1) mutations causing myotonia congenita. Neurology. (2000) 54:937–42. 10.1212/WNL.54.4.937 10690989

[101] Wu FF Ryan A Devaney J Warnstedt M Korade-Mirnics Z Poser B. Novel CLCN1 mutations with unique clinical and electrophysiological consequences. Brain. (2002) 125:2392–407. 10.1093/brain/awf246 12390967

[102] George AL Jr Sloan-Brown K Fenichel GM Mitchell GA Spiegel R Pascuzzi RM. Nonsense and missense mutations of the muscle chloride channel gene in patients with myotonia congenita. Hum Mol Genet. (1994) 3:2071–2. 7874130

[103] Meyer-Kleine C Steinmeyer K Ricker K Jentsch TJ Koch MC. Spectrum of mutations in the major human skeletal muscle chloride channel gene (CLCN1) leading to myotonia. Am J Hum Genet. (1995) 57:1325–34. 8533761 PMC1801423

[104] Macias MJ Teijido O Zifarelli G Martin P Ramirez-Espain X Zorzano A. Myotonia-related mutations in the distal C-terminus of ClC-1 and ClC-0 chloride channels affect the structure of a poly-proline helix. Biochem J. (2007) 403:79–87. 10.1042/BJ20061230 17107341 PMC1828897

[105] Raheem O Penttila S Suominen T Kaakinen M Burge J Haworth A. New immunohistochemical method for improved myotonia and chloride channel mutation diagnostics. Neurology. (2012) 79:2194–200. 10.1212/WNL.0b013e31827595e2 23152584 PMC3570820

[106] Papponen H Nissinen M Kaisto T Myllyla VV Myllyla R Metsikko K F413C and A531V but not R894X myotonia congenita mutations cause defective endoplasmic reticulum export of the muscle-specific chloride channel CLC-1. Muscle Nerve. (2008) 37:317–25. 10.1002/mus.20922 17990293

[107] Gaitan-Penas H Armand-Ugon M Macaya A Estevez R CLCN1 Myotonia congenita mutation with a variable pattern of inheritance suggests a novel mechanism of dominant myotonia. Muscle Nerve. (2018) 58:157–60. 10.1002/mus.26098 29424939

[108] Lee TT Zhang XD Chuang CC Chen JJ Chen YA Chen SC. Myotonia congenita mutation enhances the degradation of human CLC-1 chloride channels. PLoS ONE. (2013) 8:e55930. 10.1371/journal.pone.0055930 23424641 PMC3570542

[109] Desaphy JF Gramegna G Altamura C Dinardo MM Imbrici P George AL Jr. Functional characterization of ClC-1 mutations from patients affected by recessive myotonia congenita presenting with different clinical phenotypes. Exp Neurol. (2013) 248:530–40. 10.1016/j.expneurol.2013.07.018 23933576 PMC3781327

[110] Chen YA Peng YJ Hu MC Huang JJ Chien YC Wu JT. The Cullin 4A/B-DDB1-Cereblon E3 ubiquitin ligase complex mediates the degradation of CLC-1 chloride channels. Sci Rep. (2015) 5:10667. 10.1038/srep10667 26021757 PMC4448132

[111] Peng YJ Huang JJ Wu HH Hsieh HY Wu CY Chen SC. Regulation of CLC-1 chloride channel biosynthesis by FKBP8 and Hsp90β. Sci Rep. (2016) 6:32444. 10.1038/srep32444 27580824 PMC5007535

[112] Sala AJ Bott LC Morimoto RI. Shaping proteostasis at the cellular, tissue, and organismal level. J Cell Biol. (2017) 216:1231–41. 10.1083/jcb.201612111 28400444 PMC5412572

[113] Balch WE Morimoto RI Dillin A Kelly JW. Adapting proteostasis for disease intervention. Science. (2008) 319:916–9. 10.1126/science.1141448 18276881

[114] Apaja PM Lukacs GL. Protein homeostasis at the plasma membrane. Physiology. (2014) 29:265–77. 10.1152/physiol.00058.2013 24985330 PMC4103059

[115] Arvan P Zhao X Ramos-Castaneda J Chang A. Secretory pathway quality control operating in Golgi, plasmalemmal, and endosomal systems. Traffic. (2002) 3:771–80. 10.1034/j.1600-0854.2002.31102.x 12383343

[116] Saibil H. Chaperone machines for protein folding, unfolding and disaggregation. Nat Rev Mol Cell Biol. (2013) 14:630–42. 10.1038/nrm3658 24026055 PMC4340576

[117] Braakman I Hebert DN. Protein folding in the endoplasmic reticulum. Cold Spring Harb Perspect Biol. (2013) 5:a013201. 10.1101/cshperspect.a013201 23637286 PMC3632058

[118] Hebert DN Molinari M. In and out of the ER: protein folding, quality control, degradation, and related human diseases. Physiol Rev. (2007) 87:1377–408. 10.1152/physrev.00050.2006 17928587

[119] Araki K Nagata K. Protein folding and quality control in the ER. Cold Spring Harb Perspect Biol. (2011) 3:a007526. 10.1101/cshperspect.a007526 21875985 PMC3220362

[120] Ruggiano A Foresti O Carvalho P. Quality control: ER-associated degradation: protein quality control and beyond. J Cell Biol. (2014) 204:869–79. 10.1083/jcb.201312042 24637321 PMC3998802

[121] Preston GM Brodsky JL. The evolving role of ubiquitin modification in endoplasmic reticulum-associated degradation. Biochem J. (2017) 474:445–69. 10.1042/BCJ20160582 28159894 PMC5425155

[122] Sun Z Brodsky JL. Protein quality control in the secretory pathway. J Cell Biol. (2019) 218:3171–87. 10.1083/jcb.201906047 31537714 PMC6781448

[123] Derby MC Gleeson PA. New insights into membrane trafficking and protein sorting. Int Rev Cytol. (2007) 261:47–116. 10.1016/S0074-7696(07)61002-X 17560280

[124] Chia PZ Gleeson PA. Membrane tethering. F1000Prime Rep. (2014) 6:74. 10.12703/P6-74 25343031 PMC4166942

[125] Cobbold C Monaco AP Sivaprasadarao A Ponnambalam S. Aberrant trafficking of transmembrane proteins in human disease. Trends Cell Biol. (2003) 13:639–47. 10.1016/j.tcb.2003.10.008 14624842

[126] Gomez-Navarro N Miller E. Protein sorting at the ER-Golgi interface. J Cell Biol. (2016) 215:769–78. 10.1083/jcb.201610031 27903609 PMC5166505

[127] Babst M Quality control: quality control at the plasma membrane: one mechanism does not fit all. J Cell Biol. (2014) 205:11–20. 10.1083/jcb.201310113 24733583 PMC3987138

[128] Foot N Henshall T Kumar S. Ubiquitination and the regulation of membrane proteins. Physiol Rev. (2017) 97:253–81. 10.1152/physrev.00012.2016 27932395

[129] MacGurn JA Hsu PC Emr SD. Ubiquitin and membrane protein turnover: from cradle to grave. Annu Rev Biochem. (2012) 81:231–59. 10.1146/annurev-biochem-060210-093619 22404628

[130] Powers ET Morimoto RI Dillin A Kelly JW Balch WE. Biological and chemical approaches to diseases of proteostasis deficiency. Annu Rev Biochem. (2009) 78:959–91. 10.1146/annurev.biochem.052308.114844 19298183

[131] Labbadia J Morimoto RI. The biology of proteostasis in aging and disease. Annu Rev Biochem. (2015) 84:435–64. 10.1146/annurev-biochem-060614-033955 25784053 PMC4539002

[132] Hipp MS Park SH Hartl FU. Proteostasis impairment in protein-misfolding and -aggregation diseases. Trends Cell Biol. (2014) 24:506–14. 10.1016/j.tcb.2014.05.003 24946960

[133] Guerriero CJ Brodsky JL. The delicate balance between secreted protein folding and endoplasmic reticulum-associated degradation in human physiology. Physiol Rev. (2012) 92:537–76. 10.1152/physrev.00027.2011 22535891 PMC4162396

[134] Okiyoneda T Apaja PM Lukacs GL. Protein quality control at the plasma membrane. Curr Opin Cell Biol. (2011) 23:483–91. 10.1016/j.ceb.2011.04.012 21571517 PMC3148424

[135] Lukacs GL Verkman AS. CFTR: folding, misfolding and correcting the DeltaF508 conformational defect. Trends Mol Med. (2012) 18:81–91. 10.1016/j.molmed.2011.10.003 22138491 PMC3643519

[136] Foo B Williamson B Young JC Lukacs G Shrier A. hERG quality control and the long QT syndrome. J Physiol. (2016) 594:2469–81. 10.1113/JP270531 26718903 PMC4850197

[137] Ma D Jan LY. ER transport signals and trafficking of potassium channels and receptors. Curr Opin Neurobiol. (2002) 12:287–92. 10.1016/S0959-4388(02)00319-7 12049935

[138] Vandenberghe W Bredt DS. Early events in glutamate receptor trafficking. Curr Opin Cell Biol. (2004) 16:134–9. 10.1016/j.ceb.2004.01.003 15196555

[139] Nufer O Guldbrandsen S Degen M Kappeler F Paccaud JP Tani K. Role of cytoplasmic C-terminal amino acids of membrane proteins in ER export. J Cell Sci. (2002) 115:619–28. 11861768 10.1242/jcs.115.3.619

[140] Wang X Matteson J An Y Moyer B Yoo JS Bannykh S. COPII-dependent export of cystic fibrosis transmembrane conductance regulator from the ER uses a di-acidic exit code. J Cell Biol. (2004) 167:65–74. 10.1083/jcb.200401035 15479737 PMC2172508

[141] Gurnett CA Kahl SD Anderson RD Campbell KP. Absence of the skeletal muscle sarcolemma chloride channel ClC-1 in myotonic mice. J Biol Chem. (1995) 270:9035–8. 10.1074/jbc.270.16.9035 7721815

[142] Klocke R Steinmeyer K Jentsch TJ Jockusch H. Role of innervation, excitability, and myogenic factors in the expression of the muscular chloride channel ClC-1. A study on normal and myotonic muscle. J Biol Chem. (1994) 269:27635–9. 7961681

[143] Aromataris EC Rychkov GY. ClC-1 chloride channel: matching its properties to a role in skeletal muscle. Clin Exp Pharmacol Physiol. (2006) 33:1118–23. 10.1111/j.1440-1681.2006.04502.x 17042925

[144] Hutt DM Balch WE. Expanding proteostasis by membrane trafficking networks. Cold Spring Harb Perspect Biol. (2013) 5:a013383. 10.1101/cshperspect.a013383. 23426524 PMC3685892

[145] Glickman MH Ciechanover A. The ubiquitin-proteasome proteolytic pathway: destruction for the sake of construction. Physiol Rev. (2002) 82:373–428. 10.1152/physrev.00027.2001 11917093

[146] Kleiger G Mayor T. Perilous journey: a tour of the ubiquitin-proteasome system. Trends Cell Biol. (2014) 24:352–9. 10.1016/j.tcb.2013.12.003 24457024 PMC4037451

[147] Abriel H Staub O. Ubiquitylation of ion channels. Physiology. (2005) 20:398–407. 10.1152/physiol.00033.2005 16287989

[148] Deshaies RJ Joazeiro CA. RING domain E3 ubiquitin ligases. Annu Rev Biochem. (2009) 78:399–434. 10.1146/annurev.biochem.78.101807.093809 19489725

[149] Rotin D Kumar S. Physiological functions of the HECT family of ubiquitin ligases. Nat Rev Mol Cell Biol. (2009) 10:398–409. 10.1038/nrm2690 19436320

[150] Jackson S Xiong Y. CRL4s: the CUL4-RING E3 ubiquitin ligases. Trends Biochem Sci. (2009) 34:562–70. 10.1016/j.tibs.2009.07.002 19818632 PMC2783741

[151] Petroski MD Deshaies RJ. Function and regulation of cullin-RING ubiquitin ligases. Nat Rev Mol Cell Biol. (2005) 6:9–20. 10.1038/nrm1547 15688063

[152] Hannah J Zhou P. Distinct and overlapping functions of the cullin E3 ligase scaffolding proteins CUL4A and CUL4B. Gene. (2015) 573:33–45. 10.1016/j.gene.2015.08.064 26344709 PMC5110433

[153] Blondelle J Shapiro P Domenighetti AA Lange S. Cullin E3 ligase activity is required for myoblast differentiation. J Mol Biol. (2017) 429:1045–66. 10.1016/j.jmb.2017.02.012 28238764 PMC5395100

[154] Gupta VA Beggs AH. Kelch proteins: emerging roles in skeletal muscle development and diseases. Skelet Muscle. (2014) 4:11. 10.1186/2044-5040-4-11 24959344 PMC4067060

[155] Kim YE Hipp MS Bracher A Hayer-Hartl M Hartl FU. Molecular chaperone functions in protein folding and proteostasis. Annu Rev Biochem. (2013) 82:323–55. 10.1146/annurev-biochem-060208-092442 23746257

[156] Houck SA Cyr DM. Mechanisms for quality control of misfolded transmembrane proteins. Biochim Biophys Acta. (2011) 1818:1108–14. 10.1016/j.bbamem.2011.11.007 22100602 PMC3288195

[157] Taipale M Jarosz DF Lindquist S. HSP90 at the hub of protein homeostasis: emerging mechanistic insights. Nat Rev Mol Cell Biol. (2010) 11:515–28. 10.1038/nrm2918 20531426

[158] Shirane M Nakayama KI. Inherent calcineurin inhibitor FKBP38 targets Bcl-2 to mitochondria and inhibits apoptosis. Nat Cell Biol. (2003) 5:28–37. 10.1038/ncb894 12510191

[159] Okamoto T Nishimura Y Ichimura T Suzuki K Miyamura T Suzuki T. Hepatitis C virus RNA replication is regulated by FKBP8 and Hsp90. EMBO J. (2006) 25:5015–25. 10.1038/sj.emboj.7601367 17024179 PMC1618089

[160] Peng YJ Lee YC Fu SJ Chien YC Liao YF Chen TY. FKBP8 Enhances protein stability of the CLC-1 chloride channel at the plasma membrane. Int J Mol Sci. (2018) 19:E3783. 10.3390/ijms19123783 30487393 PMC6320802

[161] Andersen G Hedermann G Witting N Duno M Andersen H Vissing J. The antimyotonic effect of lamotrigine in non-dystrophic myotonias: a double-blind randomized study. Brain. (2017) 140:2295–305. 10.1093/brain/awx192 29050397

[162] Stunnenberg BC Raaphorst J Groenewoud HM Statland JM Griggs RC Woertman W. Effect of Mexiletine on Muscle Stiffness in Patients With Nondystrophic myotonia evaluated using aggregated N-of-1 trials. JAMA. (2018) 320:2344–53. 10.1001/jama.2018.18020 30535218 PMC6583079

[163] Statland JM Bundy BN Wang Y Rayan DR Trivedi JR Sansone VA. Mexiletine for symptoms and signs of myotonia in nondystrophic myotonia: a randomized controlled trial. JAMA. (2012) 308:1357–65. 10.1001/jama.2012.12607 23032552 PMC3564227

[164] Zhao J Dupre N Puymirat J Chahine M. Biophysical characterization of M1476I, a sodium channel founder mutation associated with cold-induced myotonia in French Canadians. J Physiol. (2012) 590:2629–44. 10.1113/jphysiol.2011.223461 22250216 PMC3424721

[165] Wang GK Russell C Wang SY. Mexiletine block of wild-type and inactivation-deficient human skeletal muscle hNav1.4 Na^+^ channels. J Physiol. (2004) 554:621–33. 10.1113/jphysiol.2003.054973 14608007 PMC1664796

[166] Kuo CC Lu L. Characterization of lamotrigine inhibition of Na^+^ channels in rat hippocampal neurones. Br J Pharmacol. (1997) 121:1231–8. 10.1038/sj.bjp.0701221 9249262 PMC1564785

[167] Xie X Lancaster B Peakman T Garthwaite J. Interaction of the antiepileptic drug lamotrigine with recombinant rat brain type IIA Na^+^ channels and with native Na^+^ channels in rat hippocampal neurones. Pflugers Arch. (1995) 430:437–46. 10.1007/BF00373920 7491269

[168] Brownell JE Sintchak MD Gavin JM Liao H Bruzzese FJ Bump NJ. Substrate-assisted inhibition of ubiquitin-like protein-activating enzymes: the NEDD8 E1 inhibitor MLN4924 forms a NEDD8-AMP mimetic *in situ*. Mol Cell. (2010) 37:102–11. 10.1016/j.molcel.2009.12.024 20129059

[169] Soucy TA Smith PG Milhollen MA Berger AJ Gavin JM Adhikari S. An inhibitor of NEDD8-activating enzyme as a new approach to treat cancer. Nature. (2009) 458:732–6. 10.1038/nature07884 19360080

[170] Bulatov E Ciulli A. Targeting Cullin-RING E3 ubiquitin ligases for drug discovery: structure, assembly and small-molecule modulation. Biochem J. (2015) 467:365–86. 10.1042/BJ20141450 25886174 PMC4403949

[171] McMillin DW Jacobs HM Delmore JE Buon L Hunter ZR Monrose V. Molecular and cellular effects of NEDD8-activating enzyme inhibition in myeloma. Mol Cancer Ther. (2012) 11:942–51. 10.1158/1535-7163.MCT-11-0563 22246439 PMC3755358

[172] Tanaka T Nakatani T Kamitani T. Inhibition of NEDD8-conjugation pathway by novel molecules: potential approaches to anticancer therapy. Mol Oncol. (2012) 6:267–75. 10.1016/j.molonc.2012.01.003 22306028 PMC3826113

[173] Soucy TA Dick LR Smith PG Milhollen MA Brownell JE. The NEDD8 conjugation pathway and its relevance in cancer biology and therapy. Genes Cancer. (2010) 1:708–16. 10.1177/1947601910382898 21779466 PMC3092238

[174] Powers MV Workman P. Targeting of multiple signalling pathways by heat shock protein 90 molecular chaperone inhibitors. Endocr Relat Cancer. (2006) 13(Suppl. 1):S125–35. 10.1677/erc.1.01324 17259553

[175] Chiosis G Rodina A Moulick K. Emerging Hsp90 inhibitors: from discovery to clinic. Anticancer Agents Med Chem. (2006) 6:1–8. 10.2174/187152006774755483 16475922

[176] Pillai RN Ramalingam SS. Heat shock protein 90 inhibitors in non-small-cell lung cancer. Curr Opin Oncol. (2014) 26:159–64. 10.1097/CCO.0000000000000047 24463348

[177] Lindquist SL Kelly JW. Chemical and biological approaches for adapting proteostasis to ameliorate protein misfolding and aggregation diseases: progress and prognosis. Cold Spring Harb Perspect Biol. (2011) 3:a004507. 10.1101/cshperspect.a004507 21900404 PMC3225948

[178] Tao YX Conn PM. Pharmacoperones as novel therapeutics for diverse protein conformational diseases. Physiol Rev. (2018) 98:697–725. 10.1152/physrev.00029.2016 29442594 PMC5966717

[179] Waza M Adachi H Katsuno M Minamiyama M Sang C Tanaka F. 17-AAG, an Hsp90 inhibitor, ameliorates polyglutamine-mediated motor neuron degeneration. Nat Med. (2005) 11:1088–95. 10.1038/nm1298 16155577

[180] Fujikake N Nagai Y Popiel HA Okamoto Y Yamaguchi M Toda T. Heat shock transcription factor 1-activating compounds suppress polyglutamine-induced neurodegeneration through induction of multiple molecular chaperones. J Biol Chem. (2008) 283:26188–97. 10.1074/jbc.M710521200 18632670 PMC3258858

[181] Lueck JD Mankodi A Swanson MS Thornton CA Dirksen RT. Muscle chloride channel dysfunction in two mouse models of myotonic dystrophy. J Gen Physiol. (2007) 129:79–94. 10.1085/jgp.200609635 17158949 PMC2151606

[182] Charlet BN Savkur RS Singh G Philips AV Grice EA Cooper TA Loss of the muscle-specific chloride channel in type 1 myotonic dystrophy due to misregulated alternative splicing. Mol Cell. (2002) 10:45–53. 10.1016/S1097-2765(02)00572-5 12150906

[183] Cho DH Tapscott SJ. Myotonic dystrophy: emerging mechanisms for DM1 and DM2. Biochim Biophys Acta. (2007) 1772:195–204. 10.1016/j.bbadis.2006.05.013 16876389

[184] Ursu SF Alekov A Mao NH Jurkat-Rott K. ClC1 chloride channel in myotonic dystrophy type 2 and ClC1 splicing *in vitro*. Acta Myol. (2012) 31:144–53. 23097607 PMC3476861

[185] Wheeler TM Lueck JD Swanson MS Dirksen RT Thornton CA. Correction of ClC-1 splicing eliminates chloride channelopathy and myotonia in mouse models of myotonic dystrophy. J Clin Invest. (2007) 117:3952–7. 10.1172/JCI33355 18008009 PMC2075481

[186] Cardani R Giagnacovo M Botta A Rinaldi F Morgante A Udd B. Co-segregation of DM2 with a recessive CLCN1 mutation in juvenile onset of myotonic dystrophy type 2. J Neurol. (2012) 259:2090–9. 10.1007/s00415-012-6462-1 22407275

[187] Suominen T Schoser B Raheem O Auvinen S Walter M Krahe R. High frequency of co-segregating CLCN1 mutations among myotonic dystrophy type 2 patients from Finland and Germany. J Neurol. (2008) 255:1731–6. 10.1007/s00415-008-0010-z 18807109 PMC4079033

[188] Miranda DR Wong M Romer SH McKee C Garza-Vasquez G Medina AC. Progressive Cl- channel defects reveal disrupted skeletal muscle maturation in R6/2 Huntington's mice. J Gen Physiol. (2017) 149:55–74. 10.1085/jgp.201611603 27899419 PMC5217084

[189] Waters CW Varuzhanyan G Talmadge RJ Voss AA. Huntington disease skeletal muscle is hyperexcitable owing to chloride and potassium channel dysfunction. Proc Natl Acad Sci USA. (2013) 110:9160–5. 10.1073/pnas.1220068110 23671115 PMC3670332

[190] du Souich P Roederer G Dufour R. Myotoxicity of statins: mechanism of action. Pharmacol Ther. (2017) 175:1–16. 10.1016/j.pharmthera.2017.02.029 28223230

[191] Camerino GM Musumeci O Conte E Musaraj K Fonzino A Barca E. Risk of myopathy in patients in therapy with statins: identification of biological markers in a pilot study. Front Pharmacol. (2017) 8:500. 10.3389/fphar.2017.00500 28798690 PMC5529355

[192] Camerino GM Bouche M De Bellis M Cannone M Liantonio A Musaraj K. Protein kinase C theta (PKCtheta) modulates the ClC-1 chloride channel activity and skeletal muscle phenotype: a biophysical and gene expression study in mouse models lacking the PKCtheta. Pflugers Arch. (2014) 466:2215–28. 10.1007/s00424-014-1495-1 24643479

[193] Pierno S Camerino GM Cippone V Rolland JF Desaphy JF De Luca A. Statins and fenofibrate affect skeletal muscle chloride conductance in rats by differently impairing ClC-1 channel regulation and expression. Br J Pharmacol. (2009) 156:1206–15. 10.1111/j.1476-5381.2008.00079.x 19220292 PMC2697730

[194] Ito T Ando H Suzuki T Ogura T Hotta K Imamura Y. Identification of a primary target of thalidomide teratogenicity. Science. (2010) 327:1345–50. 10.1126/science.1177319 20223979

[195] Kronke J Udeshi ND Narla A Grauman P Hurst SN McConkey M. Lenalidomide causes selective degradation of IKZF1 and IKZF3 in multiple myeloma cells. Science. (2014) 343:301–5. 10.1126/science.1244851 24292625 PMC4077049

[196] Lu G Middleton RE Sun H Naniong M Ott CJ Mitsiades CS. The myeloma drug lenalidomide promotes the cereblon-dependent destruction of Ikaros proteins. Science. (2014) 343:305–9. 10.1126/science.1244917 24292623 PMC4070318

[197] Holstein SA McCarthy PL. Immunomodulatory drugs in multiple myeloma: mechanisms of action and clinical experience. Drugs. (2017) 77:505–20. 10.1007/s40265-017-0689-1 28205024 PMC5705939

[198] Kortum KM Zhu YX Shi CX Jedlowski P Stewart AK. Cereblon binding molecules in multiple myeloma. Blood Rev. (2015) 29:329–34. 10.1016/j.blre.2015.03.003 25843596

[199] Reece D Kouroukis CT Leblanc R Sebag M Song K Ashkenas J. Practical approaches to the use of lenalidomide in multiple myeloma: a canadian consensus. Adv Hematol. (2012) 2012:621958. 10.1155/2012/621958 23097669 PMC3477526

[200] Liu Y Huang X He X Zhou Y Jiang X Chen-Kiang S. A novel effect of thalidomide and its analogs: suppression of cereblon ubiquitination enhances ubiquitin ligase function. FASEB J. (2015) 29:4829–39. 10.1096/fj.15-274050 26231201 PMC4653049

[201] Fischer ES Bohm K Lydeard JR Yang H Stadler MB Cavadini S. Structure of the DDB1-CRBN E3 ubiquitin ligase in complex with thalidomide. Nature. (2014) 512:49–53. 10.1038/nature13527 25043012 PMC4423819

[202] Thomassen M Hostrup M Murphy RM Cromer BA Skovgaard C Gunnarsson TP. Abundance of ClC-1 chloride channel in human skeletal muscle: fiber type specific differences and effect of training. J Appl Physiol. (2018) 125:470–8. 10.1152/japplphysiol.01042.2017 29722626

